# Increased MARCKS Activity in BRAF Inhibitor-Resistant Melanoma Cells Is Essential for Their Enhanced Metastatic Behavior Independent of Elevated WNT5A and IL-6 Signaling

**DOI:** 10.3390/cancers14246077

**Published:** 2022-12-10

**Authors:** Vikas Yadav, Njainday Jobe, Shakti Ranjan Satapathy, Purusottam Mohapatra, Tommy Andersson

**Affiliations:** 1 Cell and Experimental Pathology, Department of Translational Medicine, Lund University, Clinical Research Centre, Skåne University Hospital, SE 20213 Malmö, Sweden; 2 Department of Biotechnology, National Institute of Pharmaceutical Education & Research (NIPER), Guwahati 781101, Assam, India

**Keywords:** melanoma, BRAF inhibitor resistance, WNT5A, MARCKS, metastasis

## Abstract

**Simple Summary:**

Approximately 60% of all melanoma patients have cancer with an activating BRAF mutation that can be effectively treated with a BRAF inhibitor. Unfortunately, such treatment often leads to rapid development of BRAF inhibitor-resistance. These BRAF inhibitor-resistant melanomas are also characterized by increased progression and metastasis. The present study was undertaken to identify an effective antimetastatic therapy for these patients. We found increased activity of the cytoskeletal-associated MARCKS protein in BRAF inhibitor-resistant melanoma cells compared to BRAF inhibitor-sensitive melanoma cells and explored the mechanism behind this increased activity. We also showed that inhibition of MARCKS activity not only reduced the invasion and migration of BRAF inhibitor-resistance melanoma cells, but also their metastatic capacity. Our study reveals that MARCKS is a critical mediator of enhanced metastasis in BRAF inhibitor-resistant melanoma cells and thus a reasonable, novel antimetastatic therapeutic target in patients with BRAF inhibitor-resistant melanoma.

**Abstract:**

Treatment of melanoma with a BRAF inhibitor (BRAFi) frequently initiates development of BRAFi resistance, leading to increased tumor progression and metastasis. Previously, we showed that combined inhibition of elevated WNT5A and IL-6 signaling reduced the invasion and migration of BRAFi-resistant (BRAFi-R) melanoma cells. However, the use of a combined approach per se and the need for high inhibitor concentrations to achieve this effect indicate a need for an alternative and single target. One such target could be myristoylated alanine-rich C-kinase substrate (MARCKS), a downstream target of WNT5A in BRAFi-sensitive melanoma cells. Our results revealed that MARCKS protein expression and activity are significantly elevated in PLX4032 and PLX4720 BRAFi-R A375 and HTB63 melanoma cells. Surprisingly, neither WNT5A nor IL-6 contributed to the increases in MARCKS expression and activity in BRAFi-R melanoma cells, unlike in BRAFi-sensitive melanoma cells. However, despite the above findings, our functional validation experiments revealed that MARCKS is essential for the increased metastatic behavior of BRAFi-R melanoma cells. Knockdown of MARCKS in BRAFi-R melanoma cells caused reductions in the F-actin content and the number of filopodia-like protrusions, explaining the impaired migration, invasion and metastasis of these cells observed in vitro and in an in vivo zebrafish model. In our search for an alternative explanation for the increased activity of MARCKS in BRAFi-R melanoma cells, we found elevated basal activities of PKCα, PKCε, PKCι, and RhoA. Interestingly, combined inhibition of basal PKC and RhoA effectively impaired MARCKS activity in BRAFi-R melanoma cells. Our results reveal that MARCKS is an attractive single antimetastatic target in BRAFi-R melanoma cells.

## 1. Introduction

Melanoma is an aggressive form of cancer often associated with poor prognosis and limited survival [1]. An interesting observation is that melanoma is well-known for its high number of mutations [2,3]. The most frequently observed mutation in melanoma, present in almost 60% of melanomas, is the activating mutation V600E in the BRAF gene. This mutation is also present in other types of cancers and is a well-known oncogenic mutation that promotes cancer progression [3,4]. Consequently, numerous studies have identified different BRAF inhibitors that target this tumor-promoting mutation, many of which have been approved for the treatment of melanoma patients [5]. Clinical use of BRAF inhibitors (BRAFis) has significantly improved the overall survival of melanoma patients with tumors possessing an activating BRAF mutation. However, the relatively immediate development of resistance to these drugs is a clinical problem, in part because BRAF inhibitor-resistant (BRAFi-R) melanoma cells exhibit more progressive behavior, in particular enhanced migration, invasion and metastasis [4,6,7]. In an approach to increase the benefits of BRAFi treatment, a combined treatment with a BRAFi and a MEK inhibitor was launched and shown to prolong the response time by approximately 70%, to 9.5 months when compared to treatment with a BRAFi alone [8]. Such a combined treatment is not only given to stage IV but also to stage III melanoma patients. The basis for also treating the latter group of patients, with only regional metastatic lesions, comes from clinical studies also revealing clear beneficial effects of a combined treatment for stage III melanoma patients with a BRAF mutation [9]. 

Our ultimate goal is to determine the alteration(s) responsible for the aggressive behavior of melanoma cells with a combined resistance to a BRAF and a MEK inhibitor that is presently favored in the clinic. However, we believe that a reasonable approach is to first explore the mechanism(s) behind the aggressive behavior of BRAFi-R melanoma cells and then study how the addition of a MEK inhibitor modulates such a modification(s). Therefore, we have in the present study focused on the molecular mechanisms responsible for enhancing the invasive and metastatic properties of BRAFi-R melanoma cells [10] to possibly identify a druggable target that could effectively inhibit their invasive and metastatic behavior. In the present study, we have established BRAFi resistant cells by prolonged exposure to Vemurafenib or its analog, since melanoma cells made resistant to Vemurafenib are also resistant towards the more recent BRAFi dabrafenib and encorafenib [10]. 

In the search for the molecular mechanism(s) underlying acquired BRAFi resistance, several signaling pathways have been shown to be altered and proposed to be involved in the resistance acquisition process [2]. One can, as suggested by Alqathama [11], make an overall division of the suggested mechanisms responsible for acquired BRAFi resistance into those related to changes in extracellular signal-regulated kinase (ERK)-dependent pathways and those related to ERK-independent pathways. The first group includes changes in CRAF or COT expression and activity, whereas the second group includes increases in the expression and activity of different tyrosine kinase receptors, including platelet-derived growth factor receptor-β (PDGFR-β), the insulin-like growth factor 1 receptor (IGFR1) and the epidermal growth factor receptor (EGFR), as well as activation of the phosphoinositide 3-kinase/protein kinase B (AKT) signaling pathway. In addition to these signaling pathway alterations, increased expression of the WNT5A ligand, which is well known to promote melanoma progression [12,13], has been observed in BRAFi-R melanoma cells and in tumor tissue from a majority of patients with acquired BRAFi resistance. Taking these results together with results from in vitro experiments, it has been suggested that WNT5A participates in the development of acquired BRAFi resistance [12,13,14,15]. 

WNT5A is a key WNT-signaling family member that has been extensively studied for its role as a tumor promoter in melanoma, with a documented ability to enhance melanoma cell migration, invasion and metastasis [13,14,16]. This ability of WNT5A has been shown to be related to increased PKC activity and its ability to induce epithelial–mesenchymal transition (EMT)-like changes [17,18]. The tumor-promoting role of WNT5A was further supported by the observed correlation between WNT5A expression in primary melanoma tissue and reduced metastasis-free survival [19]. Furthermore, WNT5A expression is increased in the more aggressive BRAFi-R melanoma cells compared to their BRAFi-sensitive counterparts [13,14,20]. We found that a combined treatment with relatively high concentrations of the WNT5A antagonist Box5 and the IL-6 blocking antibody resulted in approximately 50% inhibition of BRAFi-R melanoma cell invasiveness [15]. Clearly, there is a need to identify an alternative and single regulator of the increased metastatic behavior of BRAFi-R melanoma cells that can be effectively targeted. 

Recently, we showed that the WNT5A/PKC pathway regulates BRAFi-sensitive melanoma cell invasion at least in part by its downstream activation of the protein myristoylated alanine-rich protein c-kinase substrate (MARCKS) [21]. MARCKS, traditionally a PKC substrate, is a well-conserved protein frequently expressed in various tissues and implicated in several different cellular processes [22]. Studies have revealed that increased tumor cell expression of MARCKS is associated with poor prognosis in several cancer types [23,24,25]. MARCKS is a dynamic molecule that constantly shuttles between the plasma membrane and the cytosol depending on its phosphorylation status. Unphosphorylated MARCKS resides in the plasma membrane, where it serves as a link between the plasma membrane and the actin cytoskeleton [26]. In contrast, phosphorylation and thus activation of MARCKS results in its detachment from the plasma membrane and translocation to the cytosol [26]. This translocation results in both liberation of the plasma membrane from the actin cytoskeleton and an increase in actin polymerization, both of which are initial events that lead to increased filopodia formation and migration [26,27]. To date, the role of MARCKS and its relation to WNT5A have not been explored in highly metastatic BRAFi-R melanoma cells. 

The aim of this study was to investigate whether the WNT5A downstream target MARCKS is an effective antimetastatic target in melanoma patients who have developed acquired resistance to BRAF inhibitors.

## 2. Materials and Methods

### 2.1. Melanoma Cell Line Culture

The HTB63 (Cat# ATCC-HTB-63; from a subcutaneous tissue-derived metastasis) and A375 (Cat# ATCC-CRL-1619; derived from a primary tumor) [28] cell lines were procured from ATCC (Manassas, VA, USA) and cultured in McCoy’s 5A medium and DMEM, respectively. Both media were supplemented with 10% fetal bovine serum (FBS; Sigma, Hamburg, Germany), 0.5 units mL^−1^ penicillin, 0.5 units mL^−1^ streptomycin, and 2 mM glutamine. The supplier confirmed the genetic authenticity of both cell lines, and we continually checked for mycoplasma contamination.

### 2.2. Establishment of BRAFi-R Melanoma Cell Lines

To establish the BRAF inhibitor-resistant (BRAFi-R) melanoma cell lines, we cultured the BRAFi-sensitive HTB63 and A375 cells with increasing concentrations of two different BRAF inhibitors (PLX4032 and PLX4720) over a period of 12 to 15 weeks, as described previously [15]. Vemurafenib or PLX4032 is a BRAFi approved by the FDA for treatment of patients harboring an activating mutation in the BRAF gene, whereas PLX4720 is an analogue of vemurafenib [29]. Briefly, we established BRAFi-R melanoma cells by exposing BRAFi-sensitive cells to cycles consisting of BRAFi treatment for three days, followed by incubation in normal medium for 1 day. The concentration of the BRAFi was gradually increased from 2 µM in the first four weeks to 5 µM for the next eight weeks. After completion of the twelve-week treatment cycle, cell lines were assessed for acquired BRAFi resistance by MTT and WST1 cell viability and proliferation assays. We also confirmed the establishment of resistant clones by checking the expression levels of BRAFi-R markers such as pERK1/2 and WNT5A. The cell lines exhibiting resistance to PLX4032 were named HTB63-R1 and A375-R1, whereas cell lines exhibiting resistance to PLX4720 were named HTB63-R2 and A375-R2. All BRAFi-R cell lines were stored in liquid nitrogen. Before these cells were used for any experiments, they were assessed for BRAFi resistance.

### 2.3. MTT and WST1 Cell Proliferation Assays

MTT and WST1 assays were used to analyze cell proliferation in accordance with the protocol described earlier [15,30]. Briefly, BRAFi-sensitive HTB63, BRAFi-sensitive A375, BRAFi-R HTB63 or BRAFi-R A375 melanoma cells (12,000 cells per well) were seeded in triplicate in flat-bottom 96-well tissue culture plates and allowed to adhere for 24 h. The cells were then treated with increasing concentrations (1.25, 2.5, 5, 10, and 20 µM) of BRAF inhibitors in complete medium for 72 h. After treatment, WST1 (Abcam, Cambridge, UK) or MTT (Sigma–Aldrich, Stockholm, Sweden) reagent at the desired concentration was added to each well and incubated for 1 h (WST1) or 3 h (MTT) at 37 °C, according to the manufacturer’s instructions. For MTT experiments, the violet formazan crystals formed during the incubation were dissolved using DMSO. After the incubation, the absorbance of the media was measured at 440 nm (WST1) whereas the absorption of the DMSO dissolved crystals was measured at 565 nm (MTT) in a multimode plate reader (FLUOstar Omega, BMG Labtech, Ortenberg, Germany).

### 2.4. Western Blotting

Proteins were extracted using a lysis buffer with the following composition; 1 M Tris-Hcl (pH 7.5), 0.5 M NaCl, 30 mM sodium pyrophosphate, 50 mM sodium fluoride, 0.5 M EDTA, 1.5 mM MgCl2, 10% glycerol, and 1% Triton X-100. Protein contents were estimated with a Pierce^TM^ BCA protein assay kit (Thermo Fisher Scientific, Rockford, IL, USA). Briefly, equal amounts (30–40 µg) of protein were loaded on SDS–PAGE gels, electrophoresed and transferred to PVDF membranes using a Trans-Blot Turbo semi-dry transfer machine (Bio-Rad Laboratories, Hercules, CA, USA). The membranes were blocked with BSA (1% w/vol) or skim milk (1% w/vol) and incubated overnight with primary antibodies as follows: anti-phospho-Thr202/Tyr204-ERK1/2 (1:1000), anti-ERK1/2 (1:1000), anti-phospho-Ser-159/163-MARCKS (1:1000), anti-PKCι (1:1000), anti-phospho-Thr-638/641-PKCα (1:1000, Cell Signaling, Danvers, MA, USA); anti-WNT5A (1:100, R&D systems); anti-phospho-Ser-729-PKCε (1:1000), anti-phospho-T555/563-PKCι (1:1000, Abcam, Cambridge, UK); anti-PKCα (1:500), anti-α-tubulin (1:10,000), anti-RhoA (1:1000), anti-PKCε (1:500), anti-MARCKS (1:500, Santa Cruz Biotechnology, Dallas, TX, USA). After washing, the membranes were incubated with an appropriate HRP-conjugated anti-rabbit, anti-goat or anti-mouse secondary antibody (1:10,000, Dako, Glostrup, Denmark) for 1 h on a shaker. After washing, protein bands were visualized on a ChemiDoc^TM^ imaging system (Bio-Rad Laboratories, Hercules, CA, USA) using Immobilon chemiluminescence substrate (Millipore, Danvers, MA, USA). Densitometric quantification of protein bands was performed using Image Lab Software (version 6.1, Bio-Rad Laboratories, Hercules, CA, USA).

### 2.5. Migration and Invasion Assays

As described previously, transwell migration and invasion assays were performed to quantify the metastatic behavior of BRAFi-R cells compared to their BRAFi-sensitive counterparts or MARCKS knockdown cells [21]. Briefly, cells were trypsinized and seeded at a number of 40,000 (invasion) or 30,000 (migration) in the upper compartments (24-well migration/invasion inserts, Corning, Bedford, MA, USA) in medium supplemented with 1% FBS. Normal growth medium containing 10% FBS was added to the lower compartments to enhance migration/invasion. After 24 h of incubation, migrated or invaded cells were fixed with ice-cold 70% ethanol. The upper surface of the insert membrane was wiped with a cotton swab to remove non-migrated/invaded cells, whereas the cells on the lower surface of the insert membrane were stained with 0.1% (w/vol) crystal violet (Labora-Chemicon, Malmo, Sweden). Images of the outer membranes were captured, and the attached cells were counted using ImageJ software (NIH, Bethesda, MD, USA).

### 2.6. siRNA and shRNA Transfection

All siRNA transfections were performed using Lipofectamine 2000 reagent (Invitrogen, Carlsbad, CA, USA) according to the manufacturer’s protocol. Briefly, 400,000 cells per well (in a 6-well plate) were transfected with 100 nM negative control siRNA (Cat# 4390843, Invitrogen, Carlsbad, CA, USA) or gene-specific siRNA in OptiMEM, which was replaced with fresh cell culture medium after 12 h. The cells were then allowed to grow for 48 h prior to any further analysis or experiment. The siRNA oligonucleotides used in this study were as follows: anti-WNT5A siRNA (Cat# s14871 and s14872), anti-IL6 siRNA (Cat# s7311 and 7312), and anti-MARCKS siRNA (Cat# s8636 and 243176) (all from Invitrogen, Carlsbad, CA, USA); and anti-PRKCE siRNA (Cat# J-004653-07-0005, Dharmacon, Horizon Discovery, Cambridge, UK).

For shRNA transfection, 1.5 µg of negative control shRNA or MARCKS-targeting shRNA plasmids was used to transfect cells in OptiMEM for 12 h. This transfection period was followed by replacement of the medium with fresh complete DMEM cell culture medium. Transfected cells were selected by 5 days of incubation with puromycin, initiated 48 h after transfection. The shRNA plasmids targeting MARCKS (sc-35858-SH) and the negative control shRNA-A (sc-108060) were obtained from Santa Cruz Biotechnology (Dallas, TX, USA).

### 2.7. IL-6 ELISA and RhoA Activity Assay

IL-6 secretion from BRAFi-sensitive or BRAFi-R melanoma cells transfected with either negative control or IL-6-targeting siRNAs was quantified in the corresponding cell culture medium (DMEM for A375 and McCoy’s 5A for HTB63, both supplemented with 1% FBS) using an IL-6 ELISA kit (Cat# EH2IL6, Invitrogen, Carlsbad, CA, USA) as previously described [31]. For all experiments, cell culture supernatants were centrifuged at 1000× *g* for 5 min to separate the medium from any remaining cell debris, after which the resulting supernatant was stored at −80 °C for further analysis. RhoA activity in lysates from BRAFi-sensitive and BRAFi-R melanoma cells was measured using a G-LISA RhoA activation assay kit (Cat# BK124, Cytoskeleton Inc., Denver, CO, USA) according to the manufacturer’s protocol.

### 2.8. Immunofluorescence Imaging

Cytoskeletal and DAPI staining were performed with minor modifications as previously described [15,21]. Briefly, 5000 cells (A375 BRAFi-sensitive or A375-R2 cells transfected with negative control or MARCKS-targeting siRNAs) were seeded on each coverslip and allowed to adhere for 24 h. The coverslips containing the attached cells were washed three times with PBS and fixed with cold 4% paraformaldehyde for 15 min at room temperature. The cells were washed three times with PBS and incubated with blocking buffer (2% BSA + 0.1% Triton X-100 dissolved in PBS) for 1 h at room temperature. After blocking, the cells were incubated with phalloidin-TRITC (dilution 1:400; Sigma–Aldrich, Stockholm, Sweden) for 1 h at room temperature. The cells were then washed three times with PBS supplemented with Tween 20 (0.1% *v*/*v*) and counterstained with DAPI for 10 min. After the cells were washed twice with PBS supplemented with Tween 20 (0.1% *v*/*v*), the coverslips containing the stained cells were mounted on glass slides with Dako fluorescence mounting medium (Dako, Santa Clara, CA, USA) and cured overnight at 4 °C before observation. Fluorescence images were acquired with a confocal microscope (LSM 700, Carl Zeiss, Oberkochen, Germany) with a 40× and 63× oil objective. TRITC fluorescence (red) intensity measurements and filopodia counting were performed using ImageJ software (NIH ImageJ, Bethesda, MD, USA) from four independent experiments.

### 2.9. Evaluation of Tumor Cell Metastasis in a Zebrafish Model

To validate the metastatic characteristics of BRAFi-sensitive A375 and A375-R2 cells, a transgenic zebrafish xenograft model was adopted. Briefly, A375 BRAFi-sensitive and A375-R2 cells were transfected with either negative control shRNA or MARCKS-targeting shRNA; prior to each experiment, the cells were labeled with DiI, after which approximately 700 cells were injected into the perivitelline space (PVS) of transgenic zebrafish (Tg (fli1:eGFP)) embryos at 2 days post-fertilization (dpf). Following injection of tumor cells, embryos were incubated at 35 °C for 72 h, after which all embryos were imaged for subsequent analysis of metastatic foci in the tail vein region. The metastatic foci were counted, and the mean fluorescence intensity (MFI) of each image was measured with ImageJ software (NIH ImageJ, Bethesda, MD, USA) using its specific macro plugin (Macro_intensity.ijm). For MFI analysis, all images with a high background were excluded. Counting of metastatic foci and MFI analysis were performed by at least two independent persons. The experimental part was performed by BioReperia AB (Linköping, Sweden), whereas the scoring and data analysis were performed by V.Y., S.R.S., and T.A.

### 2.10. Real-Time PCR

The RNeasy Mini Kit (Cat# 74134, Qiagen, Hilden, Germany) was used to isolate total RNA from cells. Equal amounts (1 µg) of RNA were used to synthesize cDNA using random hexamer primers and the M-MuLV reverse transcriptase enzyme provided in the kit (Cat# EP0441Invitrogen, Carlsbad, CA, USA). Real-time PCR was carried out using TaqMan probes (Thermo Fisher Scientific, Rockford, IL, USA) for the following genes: GAPDH (Hs04420632_g1), PRKCA (Hs00925200_m1), PRKCE (Hs00942886_m1), PRKCD (Hs01090047_m1), and PRKCI (Hs00995852_g1). The data were analyzed after normalization to the housekeeping gene GAPDH, and fold changes were calculated using MxPro software (Agilent Technologies, Santa Clara, CA, USA).

### 2.11. Statistical Analysis

Statistical analyses were performed using GraphPad Prism version 9.2.0 (San Diego, CA, USA). All experiments were performed independently at least four times, and the data are presented as the means ± SEMs. The statistical significance of differences between multiple groups was analyzed with one-way ANOVA accompanied by Dunnett’s post hoc test when the analysis included three groups or Tukey’s post hoc test when the analysis included more than three groups. However, for the zebrafish experiments, the data did not exhibit a normal distribution for any group; thus, we used the Wilcoxon-Mann–Whitney unpaired test. A *p* value less than 0.05 was considered statistically significant.

## 3. Results

### 3.1. BRAFi-R Melanoma Cells Exhibit Enhanced MARCKS Expression and Phosphorylation of MARCKS at Serine-159 and Serine-163

**Figure 1:**
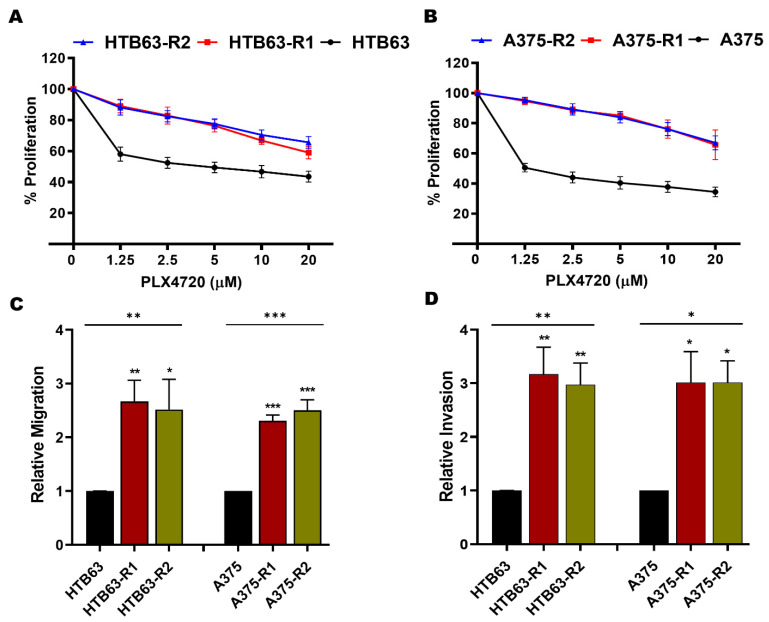
BRAFi-R melanoma cells possess higher IC_50_ value and are more aggressive than BRAFi-sensitive melanoma cells. (**A**,**B**) WST1 cell proliferation assays were performed to evaluate the development of BRAFi resistance to PLX4720 in melanoma cells, as described in Section 2. BRAFi-sensitive (black circle), PLX-4032-R (R1; red square), and PLX-4720-R (R2; blue triangle) cells were exposed to increasing concentrations of BRAF inhibitors for 72 h. Graphs were generated from 4 independent experiments, and values are presented as the means (n = 4) ± SEMs. The IC_50_ values for PLX4720 treatment were as follows: 2.5 µM for BRAFi-sensitive A375 cells, 2.5 µM for BRAFi-sensitive HTB63 cells, and >20 µM for A375-R1, A375-R2, HTB63-R1, and HTB63-R2 cells. (**C**,**D**) transwell-based migration and invasion assays were performed to evaluate the migration (**C**) and invasion (**D**) capacities of BRAFi-sensitive (HTB63 and A375), BRAFi-R1 (HTB63-R1 and A375-R1) and BRAFi-R2 (HTB63-R2 and A375-R2) melanoma cells. The numbers of migrated and invaded cells were determined using NIH ImageJ software, and the values were normalized to those in BRAFi-sensitive melanoma cells and are given as the mean ± SEM of 4 independent experiments. Statistical significance was estimated using ANOVA with Dunnett’s post hoc test for multiple comparisons; * *p* < 0.05, ** *p* < 0.01, *** *p* < 0.001.

Previously, we have shown that MARCKS activation is required for WNT5A-induced invasion of BRAFi-sensitive, melanoma cells [21]. Taken together with the observation that WNT5A expression is significantly upregulated in BRAFi-R melanoma cells [12,13,14], we sought to investigate whether WNT5A-MARCKS signaling contributes to the increased invasiveness of BRAFi-R melanoma cells. Consequently, we established BRAFi-R melanoma cell lines by gradually exposing BRAFi-sensitive HTB63 and A375 cells to increasing concentrations of two different BRAF inhibitors (PLX4032 or PLX4720). This led to the development of two PLX4032-resistant cell lines (referred to as HTB63-R1 and A375-R1) and two PLX4720-resistant cell lines (referred to as HTB63-R2 and A375-R2). The resistance of all four BRAFi-R cell lines was confirmed by WST1 and MTT assays. The IC_50_ concentrations of the BRAFis were significantly higher in all four BRAFi-R cell lines (Figure 1A,B and Figure S1A,B) compared to their BRAFi-sensitive counterparts (Figure 1A,B and Figure S1A,B). We observed that the PLX4032-resistant cell lines (HTB63-R1 and A375-R1) were equally resistant to the other BRAF inhibitor (PLX4720) and vice versa (Figure 1A,B and Figure S1A,B). These data were qualitatively confirmed by the MTT assay (Figure S2A,B). Finally, we ascertained that the BRAFi-R cells exhibited a proliferative response even in the presence of their respective BRAFi (Figure S1C,D). BRAFi-resistant melanoma cells exhibit enhanced metastasis, which is suggested to be due to their enhanced migration and invasion properties [13,32]. In good agreement with this hypothesis, we found that our four BRAFi-R melanoma cell lines exhibited increased rates of migration and invasion compared to their BRAFi-sensitive counterparts (Figure 1C,D).

**Figure 2:**
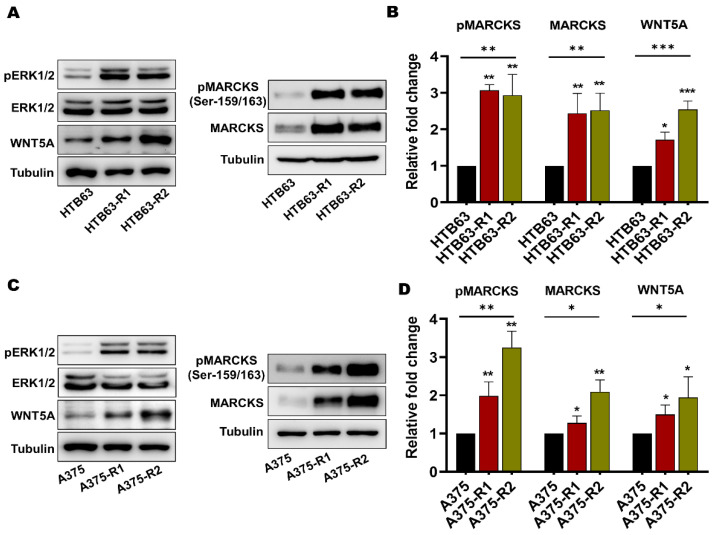
Acquired BRAFi resistance in melanoma cells causes significant increases in the levels of total MARCKS and active phospho-MARCKS. (**A**) Western blot analysis showing the endogenous levels of phospho-ERK1/2 (pERK1/2), total ERK1/2, WNT5A, phospho-MARCKS (pMARCKS; Ser-159/163), and total MARCKS in BRAFi-sensitive and BRAFi-R HTB63 cells. Tubulin was used as the loading control. Representative blots from 4 independent experiments are shown. (**B**) Graph showing the densitometric analysis of pMARCKS, total MARCKS, and WNT5A protein levels in BRAFi-sensitive and BRAFi-R HTB63 cells. The obtained values were normalized to the value of the tubulin loading control from the same sample. The calculated pMARCKS/tubulin, MARCKS/tubulin, and WNT5A/tubulin ratios were normalized to those in the BRAFi-sensitive HTB63 cells and are presented as relative fold changes. (**C**) Western blot analysis showing the endogenous levels of pERK1/2, total ERK1/2, WNT5A, pMARCKS (Ser-159/163) and total MARCKS in BRAFi-sensitive and BRAFi-R A375 cells. Tubulin was used as the loading control. Representative blots from 4 independent experiments are shown. (**D**) Graph showing the densitometric analysis of pMARCKS, total MARCKS, and WNT5A protein levels in BRAFi-sensitive and BRAFi-R A375 cells. The obtained values were normalized to the value of the tubulin loading control from the same sample. The calculated pMARCKS/tubulin, MARCKS/tubulin, and WNT5A/tubulin ratios were normalized to those in the BRAFi-sensitive A375 cells and are presented as relative fold changes. The data in the graphs (Panels B and D) were calculated from 4 independent experiments, and the results are presented as the means ± SEM. Statistical significance was estimated using ANOVA with Dunnett’s post hoc test for multiple comparisons; * *p* < 0.05, ** *p* < 0.01, *** *p* < 0.001. Original blots images can be found at File S1.

Increased MEK/ERK activity has been implicated as a marker for BRAFi resistance in melanomas [9,27]. Consequently, we validated our cells by analyzing the activating phosphorylation of ERK1/2 in the BRAFi-sensitive melanoma cells and our BRAFi-R melanoma cells by Western blotting. As shown in Figure 2, phospho-ERK1/2 levels were higher in all four BRAFi-R cells than in their BRAFi-sensitive counterparts, but no changes were observed in the levels of total ERK1/2. In addition, these cells also showed that they are able to sustain their enhanced ERK activity even in the presence of BRAFi (Figure S3A). We also compared the endogenous level of WNT5A expression in BRAFi-sensitive and BRAFi-R cells. As expected from a previous publication [15], we found a significant elevation of WNT5A expression levels in all four BRAFi-R cell lines when compared to their respective BRAFi-sensitive counterparts (Figure 2). Interestingly, the highest expression levels of WNT5A in the resistant melanoma cell lines were observed after exposure to PLX4720 (HTB63-R2 and A375-R2). Finally, we determined the levels of total MARCKS expression and active MARCKS, as indicated by its phosphorylation on Serine-159 and Serine-163, in BRAFi-sensitive and BRAFi-R HTB63 and A375 melanoma cells. As shown in Figure 2, we observed, for the first time, significant increases in the levels of total MARCKS and phosphorylated (Ser-159/163) MARCKS in all four BRAFi-R melanoma cell lines when compared to their BRAFi-sensitive counterparts, suggesting a possible role for WNT5A-MARCKS signaling in mediating the increase in the invasive and metastatic behavior of BRAFi-R melanoma cells (Figure 1C,D and Figure 2). Finally, we could also show that the BRAFi-R cells are able to sustain their enhanced MARCKS expression and its phosphorylation (Ser-159/163) even in the presence of their respective BRAFi (Figure S3B).

### 3.2. Enhanced MARCKS Expression and Activity Are Independent of WNT5A and IL-6 in BRAFi-R Melanoma Cells

**Figure 3:**
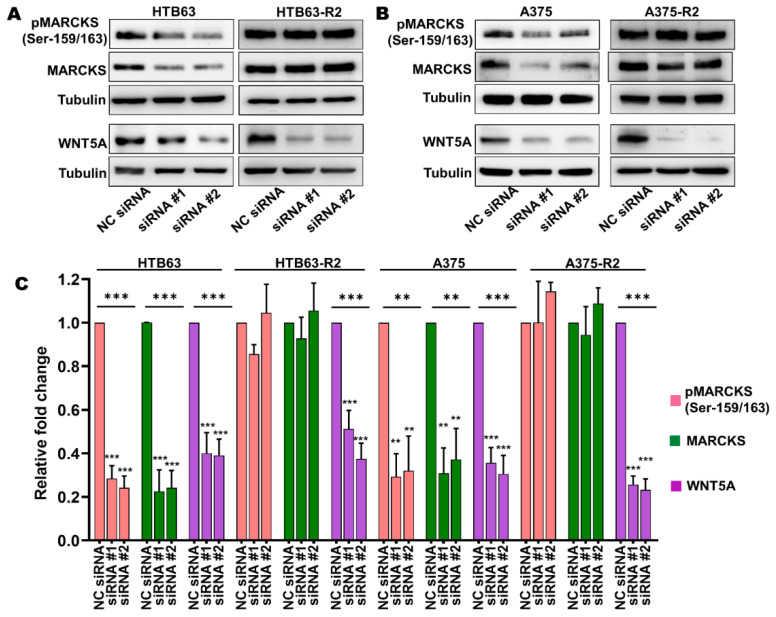
The increased levels of total MARCKS and active phospho-MARCKS are independent of the elevated WNT5A expression in BRAFi-R melanoma cells. (**A**,**B**) Western blot analysis showing the levels of pMARCKS (Ser-159/163), total MARCKS and WNT5A in BRAFi-sensitive HTB63 and BRAFi-R HTB63-R2 cells (**A**), and in BRAFi-sensitive A375 and BRAFi-R A375-R2 cells (**B**) transfected with negative control siRNA (NC; 50 nM) or either of two different WNT5A-targeting siRNAs (50 nM). Representative blots from 4 independent experiments are shown with tubulin as the loading control. (**C**) Graph showing the densitometric analysis of pMARCKS, total MARCKS, and WNT5A levels in BRAFi-sensitive and BRAFi-R2 HTB63 and A375 cells. The obtained values were normalized to the value of the tubulin loading control from the same sample. The calculated pMARCKS/tubulin, total MARCKS/tubulin, and WNT5A/tubulin ratios were normalized to those in the corresponding negative control (NC) siRNA-transfected cells and are presented as relative fold changes. The data in Panel C were calculated from 4 independent experiments, and the results are given as the means ± SEM. Statistical significance was estimated using ANOVA with Dunnett’s post hoc test for multiple comparisons; ** *p* < 0.01, *** *p* < 0.001.

The above findings led us to hypothesize that the observed increase in the expression of WNT5A could be responsible for the elevation in MARCKS activity and essential for the increased migration and invasion of BRAFi-R melanoma cells. To test this hypothesis, we decided to focus our study on PLX4720-resistant cells (HTB63-R2 and A375-R2), as these cells exhibit higher WNT5A expression levels. We reduced the expression of WNT5A by transiently transfecting BRAFi-sensitive and BRAFi-R2 melanoma cells with two independent WNT5A-targeting siRNA oligonucleotides. As shown in Figure 3, WNT5A silencing resulted in significant decreases in the protein levels of both total MARCKS and its active phosphorylated (Ser-159/163) form in BRAFi-sensitive melanoma cells, but no reductions were observed in either of the two BRAFi-R2 melanoma cell lines (Figure 3).

**Figure 4:**
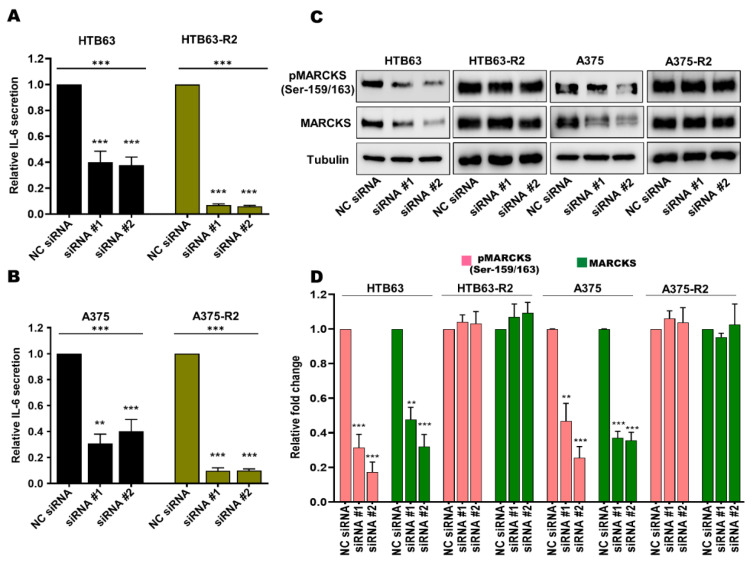
The increased levels of total MARCKS and active phospho-MARCKS are independent of the elevated IL-6 secretion in BRAFi-R melanoma cells. (**A**,**B**) ELISA-based analysis showing the secreted IL-6 level in BRAFi-sensitive HTB63 and BRAFi-R HTB63-R2 cells (**A**), and in BRAFi-sensitive A375 and BRAFi-R A375-R2 cells (**B**) transfected with negative control siRNA (NC; 50 nM) or either of two different IL-6-targeting siRNAs (50 nM). The secreted IL-6 levels were normalized to those in NC siRNA-transfected cells, and all cumulative data are presented as the means (n = 4) ± SEMs. Significance was estimated using ANOVA with Dunnett’s post hoc test for multiple comparisons; ** *p* < 0.01, *** *p* < 0.001. (**C**,**D**) Western blot analysis showing the levels of pMARCKS (Ser-159/163) and total MARCKS in BRAFi-sensitive HTB63 and BRAFi-R HTB63-R2 cells (A) and in BRAFi-sensitive A375 and BRAFi-R A375-R2 cells (**C**) transfected with negative control siRNA (NC; 50 nM) or either of two different IL-6-targeting siRNAs (50 nM). Representative blots from 4 independent experiments are shown with tubulin as the loading control. (**D**) Graph showing the densitometric analysis of the pMARCKS and total MARCKS levels in BRAFi-sensitive and BRAFi-R2 HTB63 and A375 cells. The obtained values were normalized to the value of the tubulin loading control from the same sample. The calculated pMARCKS/tubulin and total MARCKS/tubulin ratios were normalized to those in the corresponding negative control (NC) siRNA control cells and are presented as relative fold changes. The data in Panel C were calculated from 4 independent experiments, and the results are presented as the means ± SEM. Statistical significance was estimated using ANOVA with Dunnett’s post hoc test for multiple comparisons; ** *p* < 0.01, *** *p* < 0.001.

We and others have shown that in addition to WNT5A, IL-6 promotes the invasive behavior of melanoma cells [15,31,33,34,35], and since we found MARCKS activation to be independent of WNT5A, we speculated that increased IL-6 signaling could instead be responsible for promoting MARCKS activation in BRAFi-R melanoma cells. To test this hypothesis, we transiently transfected BRAFi-sensitive and BRAFi-R2 melanoma cells with two independent IL-6-targeting siRNA oligonucleotides to reduce IL-6 secretion and signaling (Figure 4A,B). IL-6 secretion from BRAFi-R2 melanoma cells was significantly reduced after transfection of the IL-6-targeting siRNAs, as was the basal level of IL-6 secretion in BRAFi-sensitive melanoma cell lines (Figure 4A,B). IL-6 knockdown in BRAFi-sensitive melanoma cells resulted in significant decreases in the protein levels of both MARCKS and phospho-MARCKS (Ser-159/163; Figure 4C,D). However, no such reductions were observed after IL-6 knockdown in BRAFi-R2 melanoma cells (Figure 4C,D). Interestingly, these results suggest that the regulatory roles of WNT5A and IL-6 in MARCKS expression and activation are lost during the acquisition of BRAFi resistance in melanoma cells.

### 3.3. MARCKS Is an Attractive Antimetastatic Target in BRAFi-R Melanoma Cells

**Figure 5:**
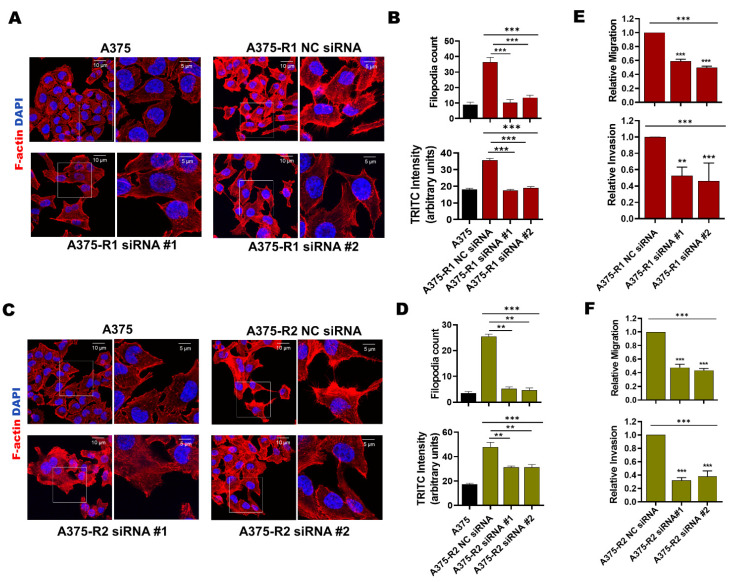
Knockdown of MARCKS is sufficient to induce morphological changes and reduce the migratory and invasive capacities of BRAFi-R melanoma cells. (**A**,**C**) A375-R1 (**A**) and A375-R2 (**C**) melanoma cells were transfected with negative control siRNA (NC; 50 nM) or either of two different MARCKS-targeting siRNAs (50 nM), after which they and A375 cells were stained with phalloidin-TRITC (F-actin) and DAPI as described in Section 2. For each image, a region of interest (marked with a white box) is further magnified and placed adjacent to its respective low magnification image. The scale bar in the lower and higher magnification images represent 10 and 5µm, respectively. (**B**,**D**) Graphs showing the mean TRITC fluorescence intensity and the number of filopodia-like protrusions in A375, A375-R1 (**B**), A375-R2 (**D**) cells transfected with control siRNA (NC; 50 nM) or either of two different MARCKS siRNAs (50 nM); both parameters were measured with NIH ImageJ software. The results are given as the means ± SEMs. (**E**,**F**) Graphs showing the migration and invasion capacities of A375-R1 (**E**) and A375-R2 cells (**F**) transfected with control siRNA (NC; 50 nM) or either of two different MARCKS siRNAs (50 nM). Migration and invasion were analyzed by transwell-based assays as described in Section 2. The numbers of migrated and invaded cells were determined using NIH ImageJ software, and the data were normalized to those in NC siRNA-transfected cells and are given as the mean ± SEM of 4 independent experiments. Statistical significance was estimated using ANOVA with Dunnett’s (**B**,**D**) and Tukey’s (**E**,**F**) post hoc tests for multiple comparisons; ** *p* < 0.01, *** *p* < 0.001.

Previous findings suggest that melanoma cells undergo major actin cytoskeleton reorganization during the acquisition of BRAFi resistance, resulting in a highly migratory and invasive cell phenotype [36]. In accordance, we observed by phalloidin-TRITC staining that in comparison with BRAFi-sensitive A375, A375-R1 and A375-R2 cells exhibited a significant increase in the amount of mean fluorescence intensity (MFI) of F-actin and number of filopodia-like membrane protrusions (Figure 5A–D). To investigate whether the increased expression and activity of MARCKS affects these invasive events in BRAFi-R melanoma cells, we performed loss-of-function experiments in BRAFi-R1 and BRAFi-R2 A375 melanoma cells by employing two independent MARCKS-targeting siRNA oligonucleotides. Both MARCKS-targeting siRNAs reduced the levels of phospho-MARCKS by 80–85% and total MARCKS by 65–70% in A375-R2 cells (Figure S4A–C). Interestingly, the MFI of F-actin and the number of filopodia-like structures were significantly reduced in A375-R1 and A375-R2 cells transfected with either of the two MARCKS siRNAs (Figure 5A–D). For clarity, we have complemented these overlay images with separate images for DAPI staining of the nuclei and phalloidin-TRITC staining of F-actin for each condition (Figure S5A,B). These results suggest that activation of MARCKS is associated with increased actin polymerization and filopodia formation at the plasma membrane in BRAFi-R cells. This process is similar to neurite formation, where MARCKS participates in F-actin-based formation of membrane protrusions [37]. In this context, we also analyzed how the migration and invasion of BRAFi-R melanoma cells were affected by knocking down MARCKS expression. We found significant reductions in the migration and invasion abilities of A375-R1, A375-R2, HTB-R1, and HTB-R2 cells transfected with MARCKS-targeting siRNAs (Figure 5E,F and Figure S6A,B). 

**Figure 6:**
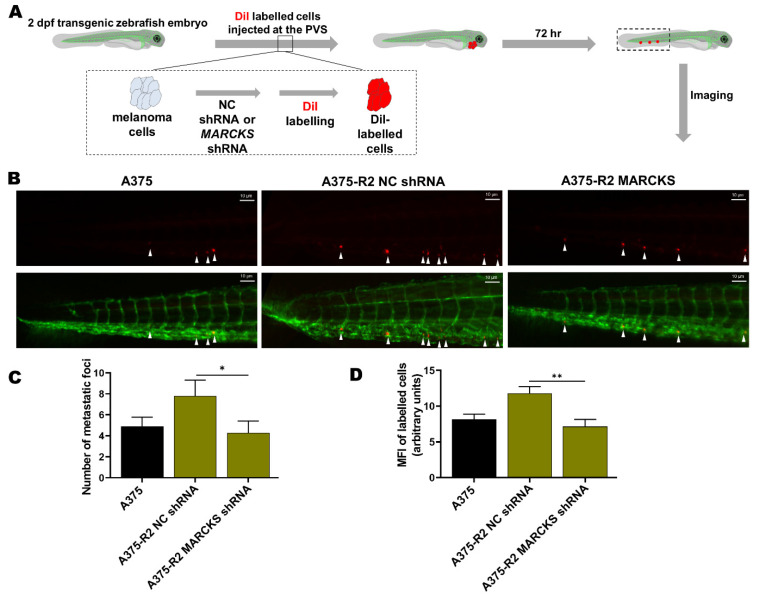
Knockdown of MARCKS is sufficient to abolish the increase in the metastatic capacity of BRAFi-R melanoma cells in a zebrafish model. (**A**) A schematic representation of the setup of the experiments with transgenic zebrafish embryos (Tg (fli1:eGFP)). (**B**) Representative images showing tail vein metastasis of BRAFi-sensitive A375 and A375-R2 cells stably transfected with either negative control shRNA or MARCKS-targeting shRNA injected into the perivitelline space (PVS) of embryos 2 days after fertilization. (**C**) Graph showing the number of metastatic foci in the tail fin region of zebrafish embryos in the BRAFi-sensitive A375 (n = 18), A375-R2 NC shRNA (n = 18), and A375-R2 MARCKS shRNA (n = 16) groups. (**D**) Graph showing the mean fluorescence intensity (MFI) of the metastatic foci in the tail fin region of zebrafish embryos in the BRAFi-sensitive A375 (n = 15), A375-R2 NC shRNA (n = 16), and A375-R2 MARCKS shRNA (n = 12) groups. Data are given as the means ± SEM. Statistical significance was estimated using an unpaired two-tailed Wilcoxon-Mann–Whitney test between the group A375-R2 NC vs. A375-R2 MARCKS sh; * *p* < 0.05, ** *p* < 0.01.

Next, we employed a zebrafish xenograft model to validate the role of MARCKS in the metastatic potential of BRAFi-R A375-R2 cells in vivo (Figure 6A). Previous studies have shown the advantages of monitoring and studying the metastatic spread of cancer cells, including melanoma cells [38,39,40]. We first transfected A375-R2 melanoma cells with either a negative control or MARCKS-targeting shRNA plasmid. The increases in the levels of phospho-MARCKS and total MARCKS in A375-R2 melanoma cells were significantly reduced after 48 h of transfection with the MARCKS-targeting shRNA vector and almost completely abolished after 144 h (Figure S7A–C). We performed in vitro migration and invasion assays to assess the efficacy of shRNA-mediated silencing of MARCKS in A375-R2 cells. We found reductions of 55% in the migration capability and 63% reduction in the invasion capability of A375-R2 cells transfected with a MARCKS-targeting shRNA vector when compared with A375-R2 cells transfected with the negative control vector (Figure S7D,E). The same passage number of NC shRNA and MARCKS shRNA transfected A375-R2 cells used above, were simultaneously injected into the zebrafish xenografts. The transgenic zebrafish embryos were divided into three separate groups and injected with either DiI-labeled BRAFi-sensitive A375 cells or DiI-labeled A375-R2 cells (transfected with either the negative control or the MARCKS shRNA vector), after which the metastatic process was analyzed as described in Section 2. A375-R2 cells transfected with negative control shRNA exhibited increased metastatic activity compared to that of BRAFi-sensitive A375 cells, as shown by either counting the metastatic foci or analyzing the MFI in the tail vein region of the zebrafish embryos (Figure 6B–D). Interestingly, transfection of A375-R2 cells with the MARCKS shRNA vector resulted in significantly fewer metastatic foci and a significantly lower MFI than those in A375-R2 cells transfected with the negative control shRNA vector. Unfortunately, it is not possible to statistically compare the metastatic activity of A375-R2 cells transfected with MARCKS shRNA with that of BRAFi-sensitive A375 melanoma cells in the present study (Figure 6B–D). Though their metastatic activities are similar, we cannot for the above reason relate this similarity to their mutual low level of MARCKS expression. 

### 3.4. BRAFi-R Melanoma Cells Exhibit Enhanced PKC Isozyme and RhoA Activities

**Figure 7:**
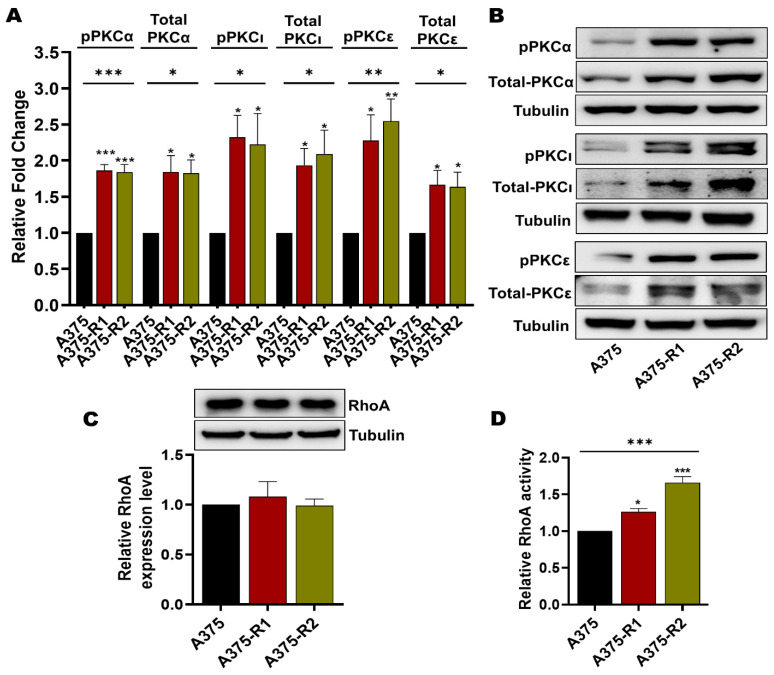
PKC and RhoA activity is enhanced in BRAFi-R melanoma cells. (**A**) Graph showing the densitometric analysis of PKCα, pPKCα, PKCι, pPKCι, PKCε, and pPKCε protein levels in BRAFi-sensitive and BRAFi-R A375 cells shown in B. These values were normalized to the value of the tubulin loading control from the same sample. The presented PKC/tubulin ratios were normalized to those in the corresponding BRAFi-sensitive cells and are given as relative fold changes. The data were calculated from 4 independent experiments and are presented as the means ± SEMs. (**B**) Western blot analysis showing the endogenous protein levels of PKCα, pPKCα (Thr-638/641), PKCι, pPKCι (T555 + 563), PKCε, and pPKCε (Ser-729) in BRAFi-sensitive and BRAFi-R A375 cells. Tubulin was used as the loading control. Representative blots from 4 independent experiments are shown. (**C**) Western blot showing RhoA protein expression in BRAFi-sensitive and BRAFi-R A375 melanoma cells. Graph below the blot show the densitometric results obtained from the value of RhoA protein expression and the tubulin loading control from the same sample. The generated RhoA/tubulin ratios were normalized to those in the corresponding BRAFi-sensitive cells and are presented as relative RhoA expression levels. The data were calculated from 4 independent experiments, and the values are presented as the means ± SEMs. (**D**) Graph showing RhoA activity in BRAFi-sensitive and BRAFi-R A375 melanoma cells as measured by a G-LISA kit. Activity levels were normalized to those in BRAFi-sensitive cells and plotted from 4 independent experiments and are given as the means ± SEM. Statistical significance was estimated using ANOVA with Dunnett’s post hoc test for multiple comparisons; * *p* < 0.05, ** *p* < 0.01, *** *p* < 0.001.

The phosphorylation and thereby the activation of MARCKS is traditionally considered to be due to PKC activity, although the literature suggests that MARCKS phosphorylation can also be induced via the RhoA/Rho-associated kinase (ROCK) signaling pathway [41,42]. This possibility is of particular interest for the present study since the WNT5A ligand has been demonstrated to activate both PKC and RhoA signaling in human melanoma cells [18,21,43]. Regarding PKC, there are nine PKC isozymes encoded by nine specific genes; however, only six have been associated with melanoma pathogenesis [44]. Of these, six PKC isozymes, only five have been coupled to increased metastasis: PKCδ, PKCα, PKCβ, PKCε, and PKCι [45,46,47,48,49,50]. In the present study, we first screened for any differences in the mRNA expression of the genes encoding these five PKC isozymes (PRKCD, PRKCA, PRKCB, PRKCE and PRKCI) between the BRAFi-sensitive and BRAFi-R melanoma cell lines. As shown in Figure S8A, A375-R1 and A375-R2 melanoma cells exhibited significantly increased expression of PRKCA, PRKCE and PRKCI compared to their BRAFi-sensitive counterpart. In contrast, we observed no difference between the mRNA level of PRKCD in BRAFi-sensitive and A375-R1 or A375-R2 melanoma cells and no detectable PRKCB mRNA in either BRAFi-sensitive A375, A375-R1 or A375-R2 melanoma cells (Figure S8A). These results suggest a possible increase in the activity of PKCα, PKCε and PKCι in A375-R1 and A375-R2 melanoma cells. To validate these mRNA expression data, we compared the protein expression levels of PKCα, PKCε and PKCι and the corresponding phosphorylation levels in BRAFi-sensitive A375, A375-R1 and A375-R2 melanoma cells by Western blotting. As shown in Figure 7A,B, the levels of PKCα, phospho-PKCα (Thr-638/641), PKCε, phospho-PKCε (Ser-729), PKCι, and phospho-PKCι (T555/563) were significantly elevated in both A375 BRAFi-R1/R2 cells when compared to their counterpart BRAFi-sensitive cells.

Our next step was to analyze the level of total RhoA in BRAFi-sensitive and BRAFi-R melanoma cells by Western blotting. Our results revealed that BRAFi-sensitive A375, A375-R1 and A375-R2 melanoma cells exhibited similar expression levels of RhoA (Figure 7C). However, when we measured RhoA activity [43], we found that both A375-R1 and A375-R2 cells exhibited significantly higher RhoA activity levels than their BRAFi-sensitive counterpart (Figure 7D).

### 3.5. Endogenous Basal PKC Activity and RhoA Activity Independently Contribute to MARCKS Activation in BRAFi-R Melanoma Cells

**Figure 8:**
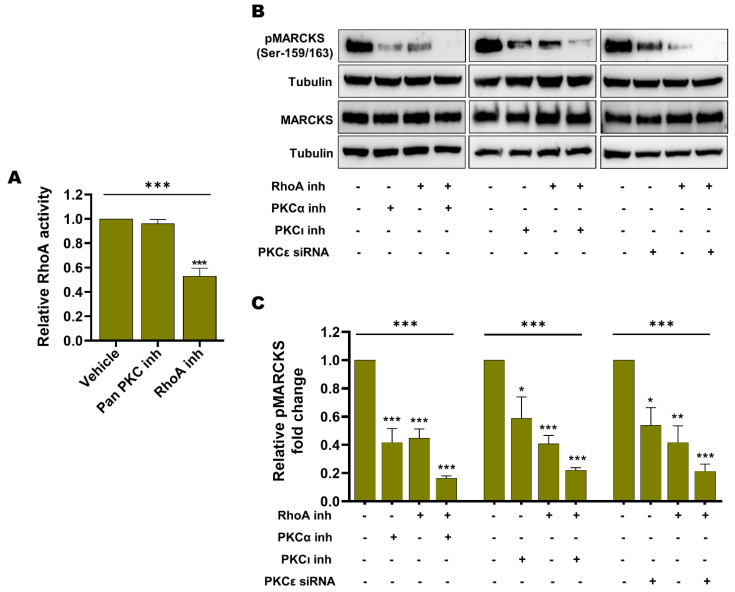
Enhanced RhoA activity and enhanced PKC activity are independent events contributing to the increased level of active phospho-MARCKS in BRAFi-R melanoma cells. (**A**) Graph showing RhoA activity as measured by a G-LISA kit in A375-R2 cells treated with either the pan-PKC inhibitor Gö6983 (50 nM) or the RhoA inhibitor Rhosin (10 μM) for 1 hr. Activity levels were normalized to those in vehicle-treated BRAFi-R A375-R2 cells and calculated from 4 independent experiments, and the values are presented as the means ± SEMs. (**B**) Western blot showing the pMARCKS (Ser-159/163) and total MARCKS levels in A375-R2 cells treated with the PKCα inhibitor (100 nM), the PKCι inhibitor (1 µM) or PKCε siRNA (100 nM) individually or in combination with the RhoA inhibitor Rhosin (100 μM). Tubulin was used as the loading control. Representative blots from 4 independent experiments are shown. (**C**) Graph showing the densitometric analysis of the pMARCKS level in A375-R2 cells as shown in panel B. These values were normalized to the value of the tubulin loading control from the same sample. The calculated pMARCKS/tubulin ratios were normalized to those in vehicle-treated BRAFi-R A375-R2 cells and are presented as relative fold changes. The data were calculated from 4 independent experiments and are presented as the means ± SEM. Statistical significance was estimated using ANOVA with Dunnett’s post hoc test for multiple comparisons; * *p* < 0.05, ** *p* < 0.01, *** *p* < 0.001.

We first sought to investigate whether RhoA activation is dependent on PKC activity, as previously suggested in fibroblasts and endothelial cells [51,52,53]. To test this hypothesis, we decided to use A375-R2 cells, as these cells exhibit a higher RhoA activity. As shown in Figure 8A, we found that the pan-PKC inhibitor Gö6983, in contrast to the RhoA inhibitor Rhosin used as a positive control, was unable to reduce RhoA activity in A375-R2 cells.

Next, we investigated the impact of specific inhibition of each of the three PKC isozymes (PKCα, PKCε and PKCι) that we showed to be upregulated and phosphorylated in BRAFi-R melanoma cells (Figure 8A–C) on MARCKS activation in A375-R2 cells. As shown in Figure 8B,C, inhibition of PKCα or PKCι alone caused a decrease of 60% and 50%, respectively, in MARCKS phosphorylation (Ser-159/163), whereas siRNA-mediated knockdown of PKCε (Figure S8B) resulted in a decrease of 50% in MARCKS phosphorylation (Ser-159/163); (Figure 8B,C). In addition, we noted that inhibition of RhoA alone resulted in a prominent reduction of 60% in MARCKS phosphorylation (Ser-159/163). Notably, inhibition of any of these three PKC isozymes (PKCα, PKCε or PKCι) in combination with treatment with the RhoA inhibitor Rhosin caused, under all three conditions, a decrease of approximately 80% in the level of phosphorylated MARCKS (Ser-159/163; Figure 8B,C). Although one cannot directly compare the effects of the different means of inhibiting the three different PKC isozymes in the present study, it is clear that combined inhibition of RhoA and any of the three PKC isozymes individually (Figure 8B,C) was the most effective means of inhibiting MARCKS activity. Notably, there was no apparent alteration in total MARCKS expression during treatment with any of the PKC isozyme inhibitors/siRNAs or the RhoA inhibitor either alone or in combination (Figure 8B). Our results indicate that the increased basal levels of RhoA and PKC signaling constitute independent mechanisms of MARCKS activation in BRAFi-R melanoma cells and that combined inhibition of RhoA and PKC signaling is an effective means of inhibiting MARCKS activity.

## 4. Discussion

Despite improvements in cancer drug discovery, the development of resistance to existing antineoplastic drugs has emerged as a major clinical problem in managing the treatment of cancer patients [54]. In melanoma, resistance to BRAF inhibitors has become one such complication. This complication is due to not only the reduced effects of BRAF inhibitors on BRAFi-R cells but also the enhanced invasive and metastatic activity of these resistant cells [12,55,56]. In the present study, we identified elevated MARCKS expression and activity as critical mediators of the increased invasive behavior and metastatic capacity of BRAFi-R melanoma cells.

Recently, we reported that the PKC substrate MARCKS is required for WNT5A-induced invasion of melanoma cells that have not been exposed to a BRAFi and consequently have not developed any resistance to the drug [21]. Here, we found that substantial increases in the levels of MARCKS expression and activating phosphorylation were correlated with elevated WNT5A expression and increased invasion and migration of BRAFi-R melanoma cells. Previous studies performed in pancreatic and hematological cancers have also revealed increased MARCKS expression and MARCKS phosphorylation in cancer cells that have developed resistance to drugs such as oxaliplatin and proteasome inhibitors [57,58,59,60]. However, none of the aforementioned studies were directed toward exploring whether and how the MARCKS protein contributes to the increased invasion and migration of drug-resistant cancer cells. In contrast to our previous findings showing a regulatory role of WNT5A in MARCKS expression and activation in BRAFi-sensitive melanoma cells, our present investigation revealed a lack of such a relationship in BRAFi-R melanoma cells. This conclusion was extended by our present results showing that siRNA-mediated knockdown of IL-6 decreased MARCKS expression and activation in BRAFi-sensitive melanoma cells, whereas IL-6 knockdown had no effects on either the elevated level of MARCKS expression or its increased activating phosphorylated status in BRAFi-R melanoma cells. These findings of altered signaling properties during development of BRAFi-R are in line with a previous report from our laboratory showing that the positive feedback signaling loop between WNT5A and IL-6 is abolished during the acquisition of BRAFi resistance [15]. Conclusively, our present results and previous data highlight that several signaling events are altered in melanoma cells during the acquisition of BRAFi resistance. We believe that further exploration of this altered signaling may identify additional therapeutic targets with the potential to interfere with the increased metastatic properties of BRAFi-R melanoma cells.

Our study highlights important regulatory roles of elevated basal RhoA and PKC activity in mediating MARCKS activation in BRAFi-R melanoma cells. Our analysis revealed that RhoA as well as three PKC isozymes (PKCα, PKCε, and PKCι) exhibited increased activity in BRAFi-R melanoma cells. Furthermore, we found that compared with individual inhibition, combined inhibition of RhoA and PKC isozymes resulted in more profound impairment of MARCKS activation in BRAFi-R cells. These findings suggest that dual targeting of RhoA and PKC isozymes could be an effective strategy to counteract MARCKS activation in BRAFi-R melanoma cells. Previously, only two studies have examined the role of RhoA and PKC activation in BRAFi resistance. In one such study, it was argued that an increase in stress fiber formation was mediated via increased RhoA activity. However, these authors did not directly analyze RhoA activity but only phosphorylated myosin light chain-2 (p-MLC2) as a presumed marker for RhoA activation in BRAFi-R melanoma cells [61]. Using this indirect approach to estimate RhoA activation, the authors found increased RhoA activity in only half of the developed BRAFi-R melanoma cell lines [61]. In another study, it was shown that the inhibition of PKCδ by a small chimeric molecule induced apoptosis in BRAFi-R melanoma cells [62]. Surprisingly, in neither of these two studies did the authors explore the relationship between RhoA and PKC in terms of their activation of MARCKS nor their possible downstream effects on the invasion, migration and metastatic capacities of BRAFi-R melanoma cells. We believe that our study is the first to define RhoA and PKC as parallel and independent regulators of MARCKS activity in BRAFi-R melanoma cells.

We next assessed the functional consequences of modulating MARCKS activity in BRAFi-R melanoma cells with the aim of effectively counteracting the increase in metastatic activity seen in these melanoma cells. We found that siRNA-mediated knockdown of MARCKS expression and activity not only reduced the F-actin content and the number of filopodia-like membrane protrusions in BRAFi-R melanoma cells but also reduced their migration and invasion abilities. In agreement with these findings, activation of MARCKS in other cell types has been reported to reduce the interaction between the plasma membrane and the actin cytoskeleton as well as induce actin polymerization, both of which are essential features for cell adhesion, spreading and subsequent migration [26,63]. Our present findings suggest that inhibition of MARCKS alone suppresses the migration and invasion of BRAFi-R melanoma cells in vitro by approximately 50%. Furthermore, our zebrafish experiments revealed that the increase in the metastatic behavior of BRAFi-R melanoma cells was abolished by single targeting of MARCKS by shRNA-mediated knockdown of MARCKS expression and activity. This observation highlights a conceivable potential of targeting MARCKS to overcome the enhanced metastasis in patients with melanoma who have acquired BRAFi resistance. 

Interestingly, as discussed in a recent review article, three different peptides have been described to have the ability to inhibit MARCKS activity [26]. These three peptides are named MARCKS ED-HIV TAT, MANS and MPS, and they impair the phosphorylation and thereby the activation of MARCKS by binding to three different domains in the MARCKS protein. Experimentally, the MARCKS ED-HIV TAT peptide has been shown to be effective in sensitizing non-small lung cancer cells to radiation therapy [64]. However, the MANS and MPS peptides have been shown to experimentally reduce the metastasis of lung cancer cells [65] and the migration of kidney cancer cells [66]. Interestingly, a search on the clinical trial homepage (www.clinicaltrials.gov; accessed on 01 07 2022) indicates that a modified acetylated MANS peptide named BIO-11006, developed by Biomarck Pharmaceuticals Ltd. (Newtown, PA, USA) has been tested in a phase II trial as a supplement to the conventional therapy in the treatment of non-small cell lung carcinoma (NCT 03472053). Additionally, the same peptide is to be tested in a phase II trial for the treatment of lung metastasis derived from Ewing’s sarcoma or osteosarcoma (NCT 04183062). In both these trials, the peptide has been aerosolized and administered through a nebulizer. Consequently, peptide inhibition of MARCKS has not been tested for preventing the metastatic spread of cancer, including drug-resistant cancer. It is therefore reasonable to presume that further experimental research is needed to identify inhibitory peptides or small molecules that systemically targets MARCKS and thus can be used to prevent metastatic disease. In summary, our study demonstrates that MARCKS is an attractive target for the future development of an effective antimetastatic compound that can counteract the increased metastatic behavior of BRAFi-R melanoma cells.

## 5. Conclusions

We reveal for the first time that MARCKS expression and activity are increased in BRAFi-R melanoma cells. Unlike in BRAFi-sensitive melanoma cells, WNT5A and IL-6 signaling have no effect on MARCKS expression and activity in BRAFi-R melanoma cells. Despite the above observations, we found that knockdown of MARCKS in BRAFi-R melanoma cells reduced their invasion and migration in vitro and decreased their metastatic behavior in an in vivo zebrafish model. In search of explanation for enhanced MARKS activity in BRAFi-R melanoma cells, we found elevated basal activities of PKCα, PKCε, PKCι, and RhoA. Taken together, these data demonstrate that the MARCKS protein is an attractive antimetastatic target for melanoma patients who have acquired BRAFi resistance.

## Data Availability

The data that support the findings of this study are available within the article or supplementary material and are also available from the corresponding author upon reasonable request.

## Supplementary Materials

The following are available online at https://www.mdpi.com/article/10.3390/cancers14246077/s1, Figure S1: WST1 assay showing development of BRAFi-R melanoma cells to BRAF inhibitors; Figure S2: MTT assay showing the development of BRAFi-R melanoma to BRAF inhibitors; Figure S3: Western blot analysis showing the levels of pERK1/2, total ERK1/2; pMARCKS, and MARCKS in A375-R1 and A375-R2 cells after treating with their respective BRAF inhibitors; Figure S4: Western blot showing the levels of pMARCKS and MARCKS in A375-R2 cells transfected with negative control or MARCKS-targeting siRNAs; Figure S5: Separate DAPI and phalloidin staining images corresponding to Figure 5A,C at 10 µm scale bars; Figure S6: transwell-chamber based assays showing the migration and invasion capacities of HTB63-R2 cells transfected with control siRNA or either of two different MARCKS-targeting siRNAs; Figure S7: (A, B, C) Western blot analysis showing the levels of pMARCKS (Ser-159/163) and total MARCKS in A375 BRAFi-sensitive cells and in non-transfected BRAFi-R A375-R2 cells as well as in BRAFi-R A375-R2 cells transfected with negative control shRNA or MARCKS-targeting shRNA, (D, E) transwell-based migration and invasion of the cells of above group; Figure S8: (A) Graph showing the relative mRNA expression of PRKCA, PRKCB, PRKCD, PRKCE, and PRKCI in BRAFi-sensitive, A375-R1 and A375-R2 cells, (B) Western blot analysis showing the levels of total PKCε in A375-R2 cells transfected with negative control siRNA and PKCε-targeting siRNA in A375-R2 cells. File S1: Original blots of Figure 2, Figure 3, Figure 4, Figure 7, Figure 8, S3, S4, S7, S8.
