# Supplementary material for: Increased MARCKS Activity in BRAF Inhibitor-Resistant Melanoma Cells Is Essential for Their Enhanced Metastatic Behavior Independent of Elevated WNT5A and IL-6 Signaling
Source: Cancers (Basel). 2022 Dec 10;14(24):6077. doi: 10.3390/cancers14246077 (PMC9775662; doi:10.3390/cancers14246077)

Increased MARCKS activity in BRAF inhibitor-resistant melanoma cells is essential for their enhanced metastatic behavior independent of elevated WNT5A and IL-6 signaling

Vikas Yadav [1,*], Njainday Jobe [1], Shakti Ranjan Satapathy [1], Purusottam Mohapatra [1,2], and Tommy Andersson [1,*]

[1] Cell and Experimental Pathology, Department of Translational Medicine, Lund University, Clinical Research Centre, Skåne University Hospital, SE 20213 Malmö, Sweden.

[2] Present Address: Department of Biotechnology, National Institute of Pharmaceutical Education & Research (NIPER), Guwahati, 781101, Assam, India

Original images of western blot experiments

Figure 2A

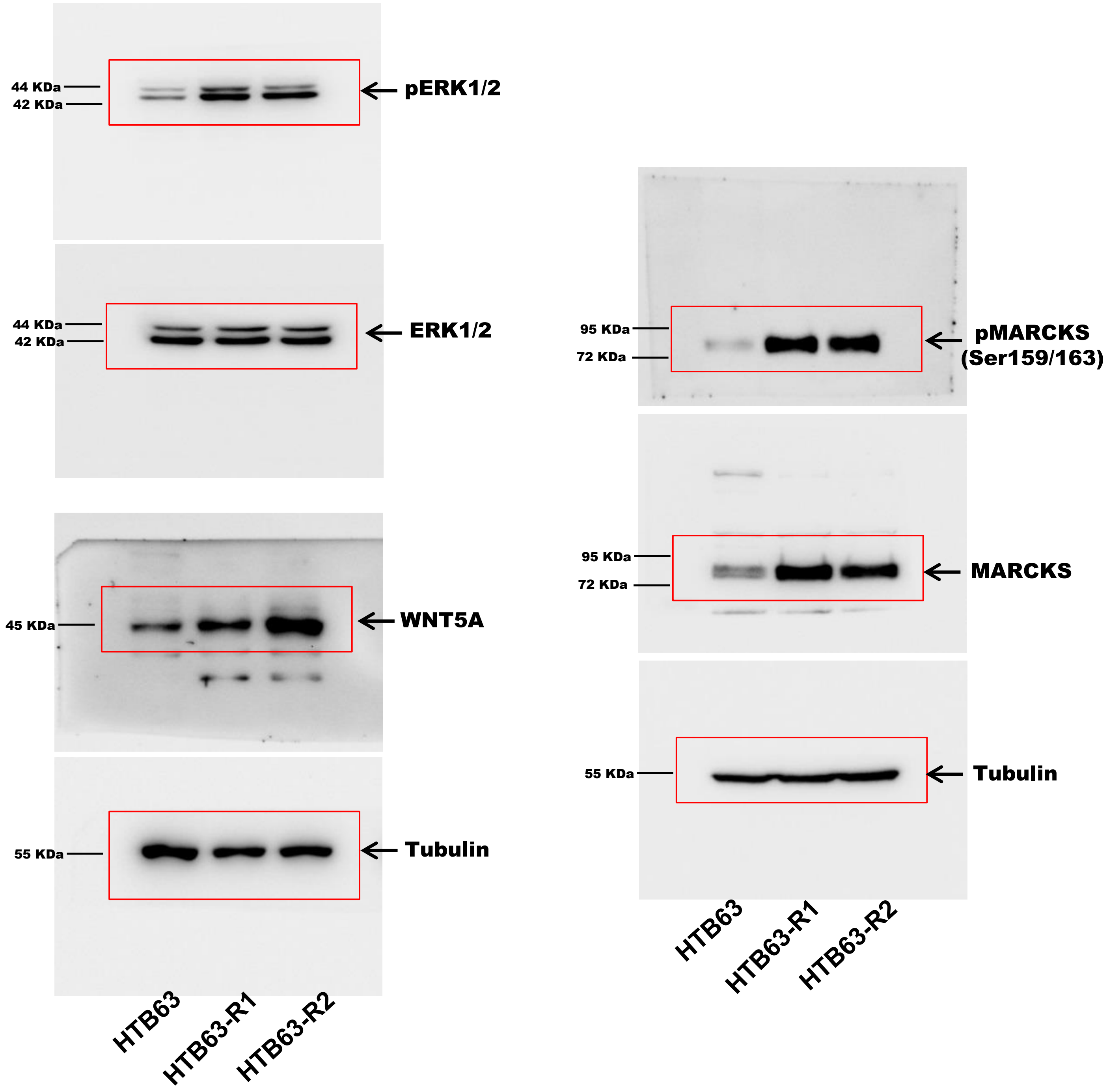
44 KDa
42 KDa
pERK1/2
44 KDa
42 KDa
ERK1/2
45 KDa
WNT5A
55 KDa
Tubulin
HTB63
HTB63-R1
HTB63-R2
95 KDa
72 KDa
pMARCKS (Ser159/163)
95 KDa
72 KDa
MARCKS
55 KDa
Tubulin
HTB63
HTB63-R1
HTB63-R2

Figure 2C

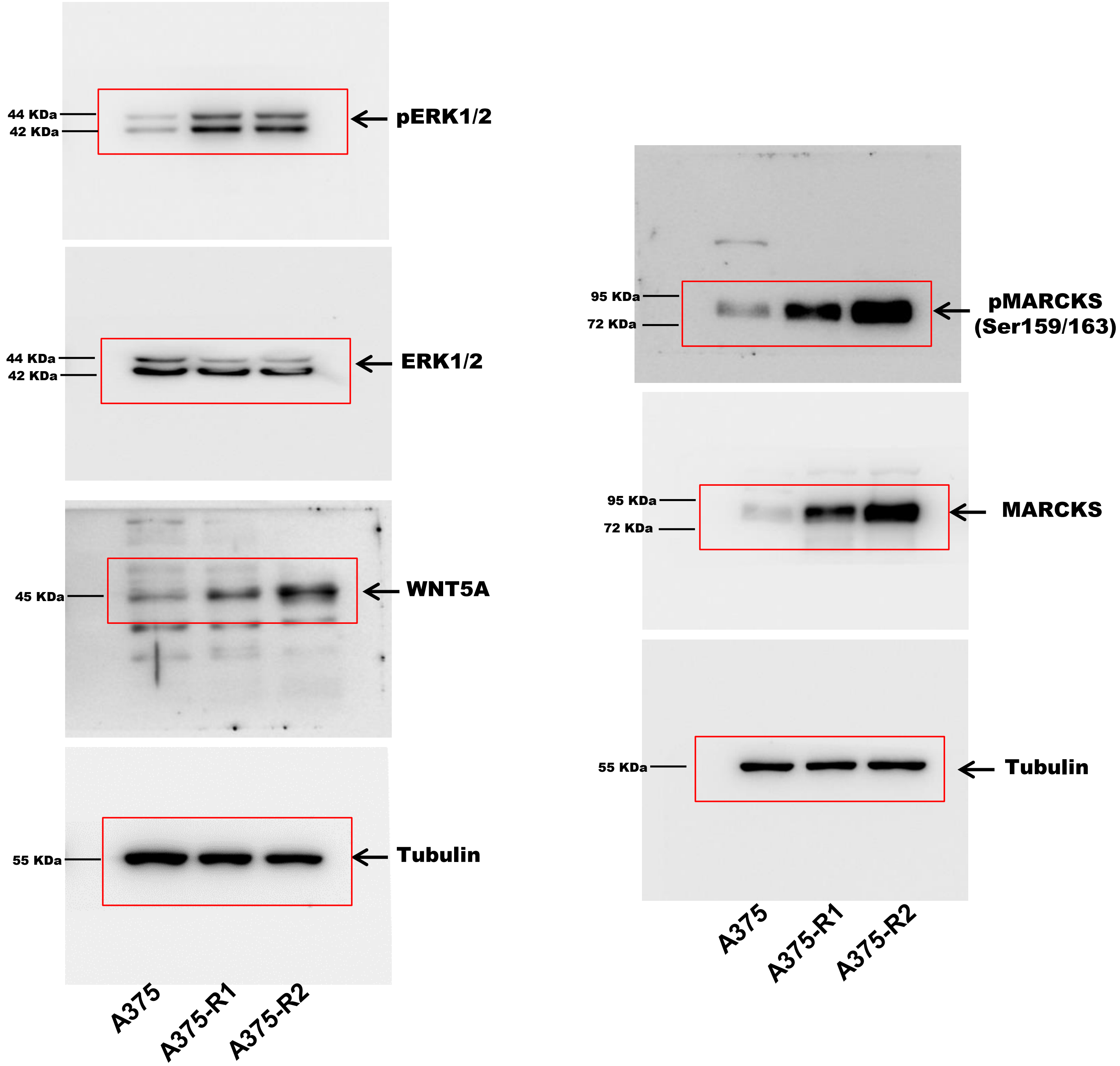
44 KDa
42 KDa
pERK1/2
44 KDa
42 KDa
ERK1/2
45 KDa
WNT5A
55 KDa
Tubulin
A375
A375-R1
A375-R2
95 KDa
72 KDa
pMARCKS (Ser159/163)
95 KDa
72 KDa
MARCKS
55 KDa
Tubulin
A375
A375-R1
A375-R2

Figure 3A

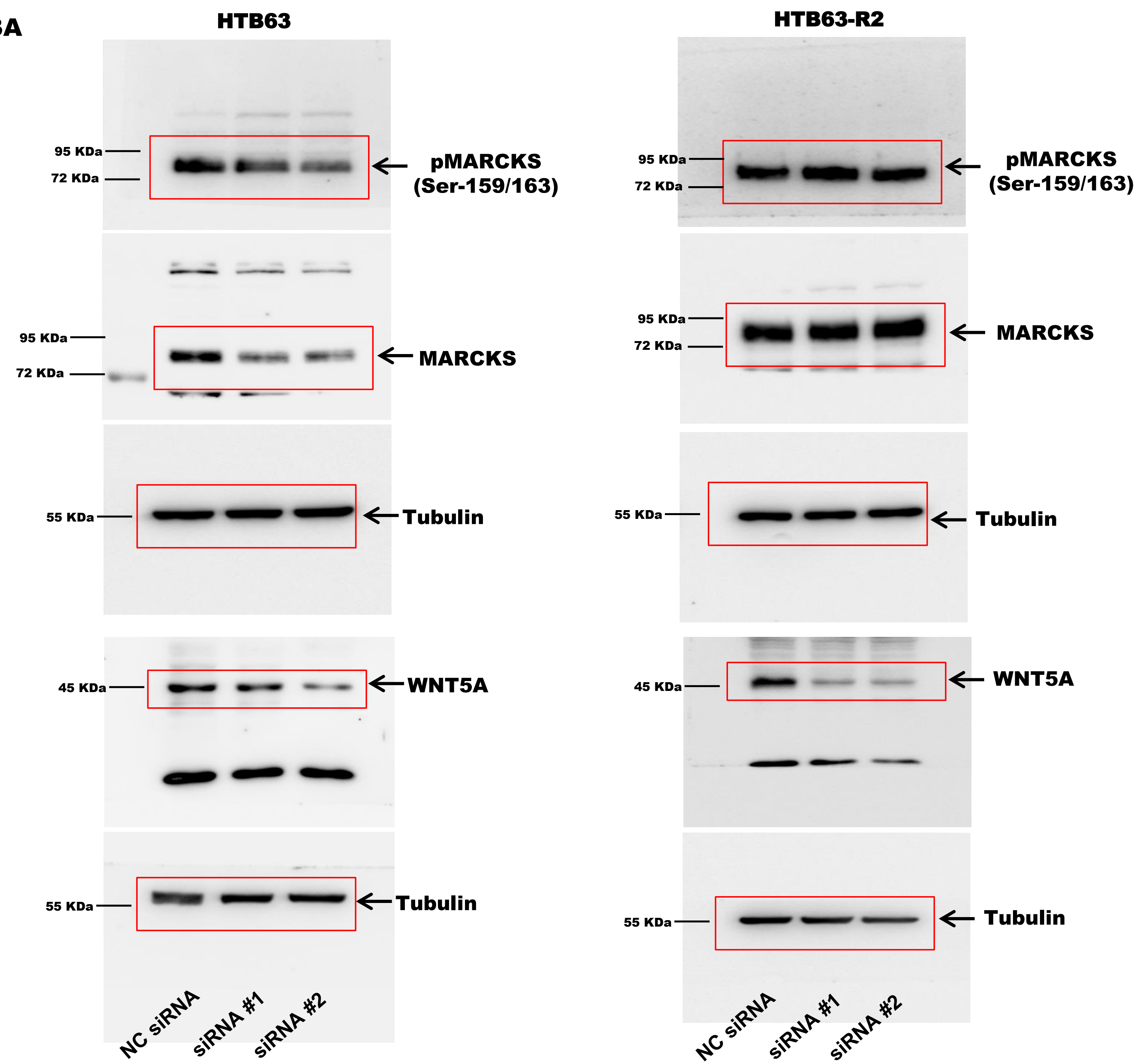

HTB63
HTB63-R2
95 KDa
72 KDa
pMARCKS (Ser-159/163)
95 KDa
72 KDa
pMARCKS (Ser-159/163)
95 KDa
72 KDa
MARCKS
95 KDa
72 KDa
MARCKS
55 KDa
Tubulin
55 KDa
Tubulin
45 KDa
WNT5A
45 KDa
WNT5A
55 KDa
Tubulin
55 KDa
Tubulin
NC siRNA
siRNA #1
siRNA #2
NC siRNA
siRNA #1
siRNA #2

Figure 3B

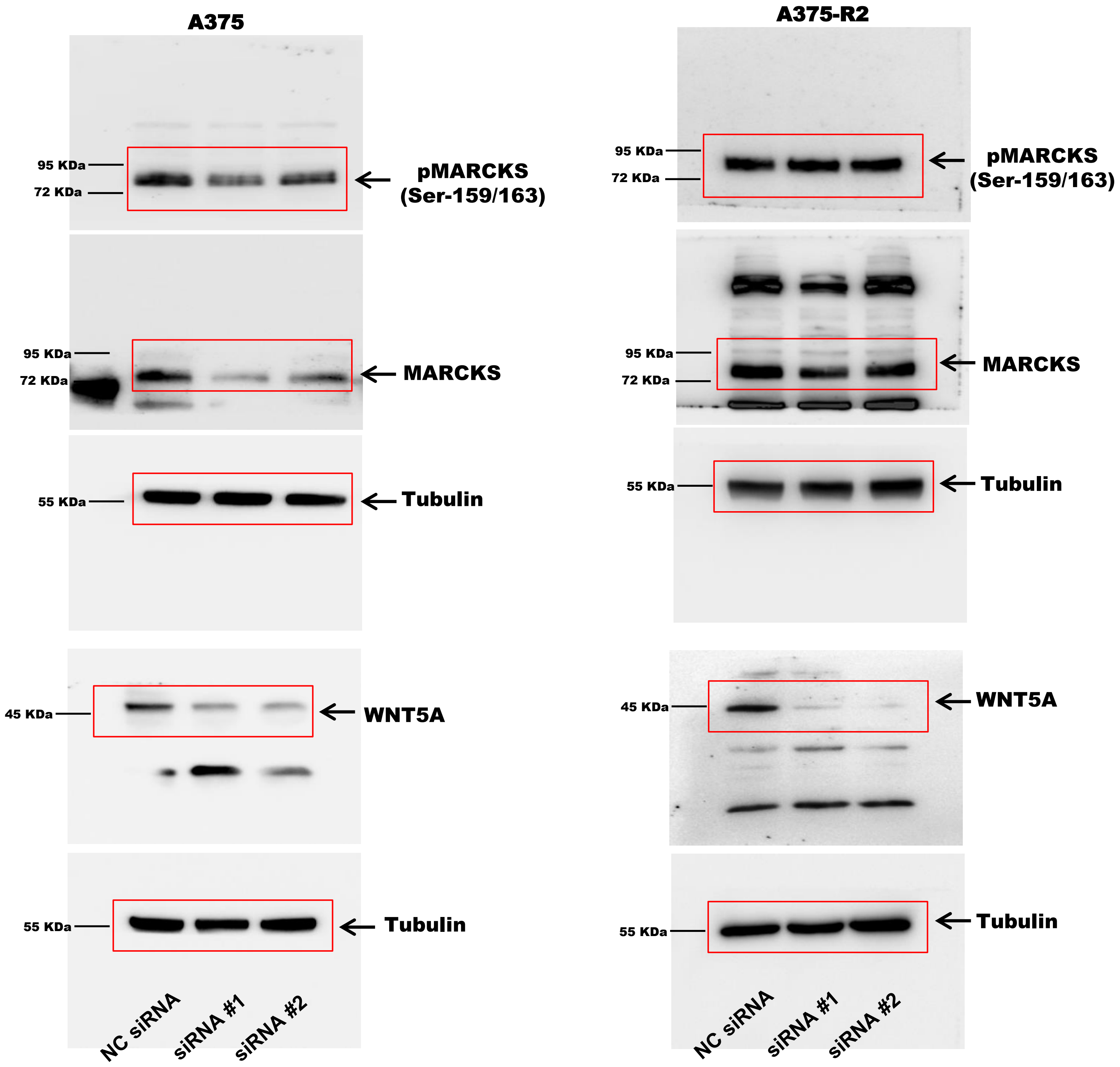
A375
A375-R2
95 KDa
72 KDa
pMARCKS
(Ser-159/163)
MARCKS
55 KDa
Tubulin
45 KDa
WNT5A
NC siRNA
siRNA #1
siRNA #2

Figure 4C

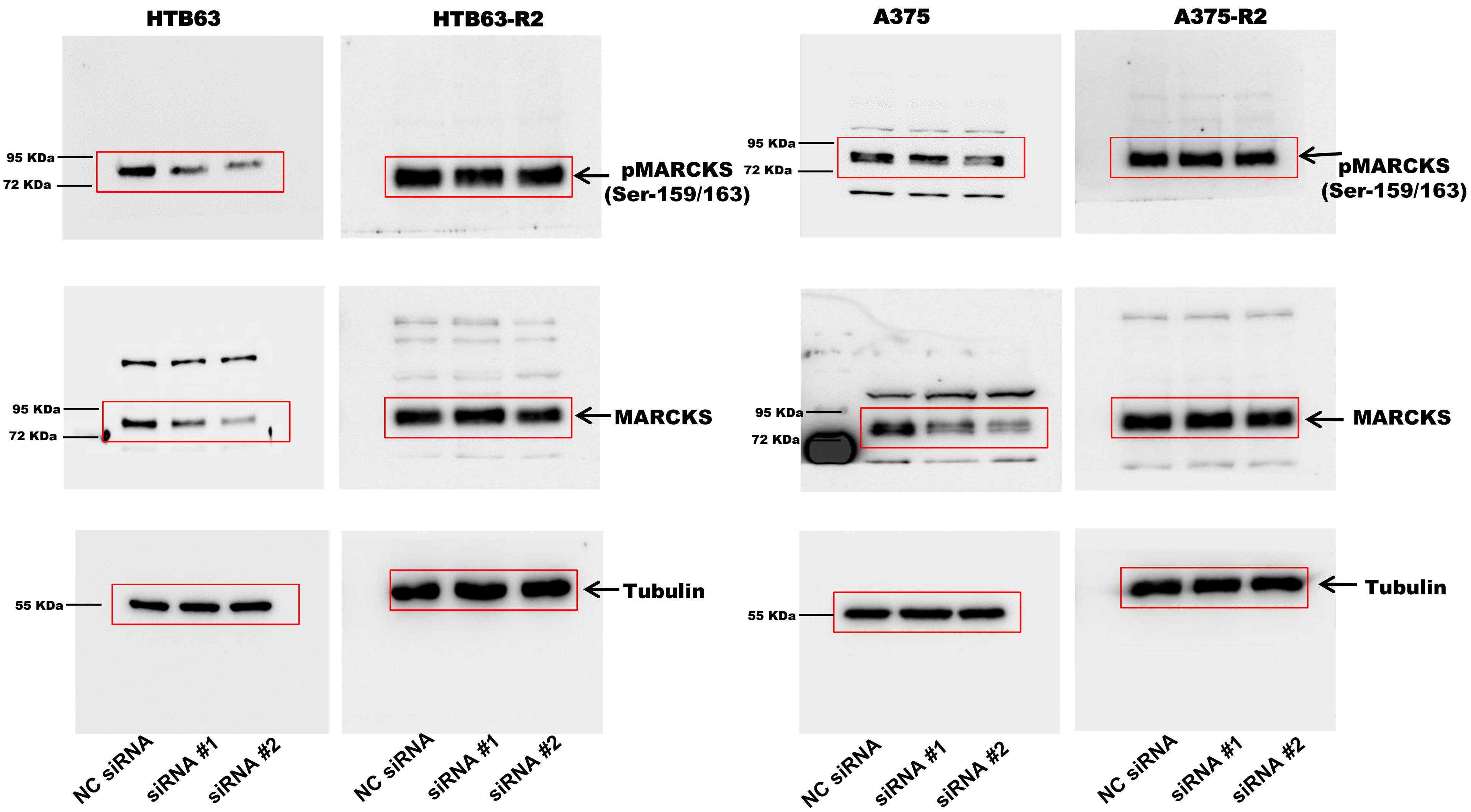
HTB63
HTB63-R2
A375
A375-R2
95 KDa
72 KDa
55 KDa
pMARCKS
(Ser-159/163)
MARCKS
Tubulin
NC siRNA
siRNA #1
siRNA #2

Figure 7B

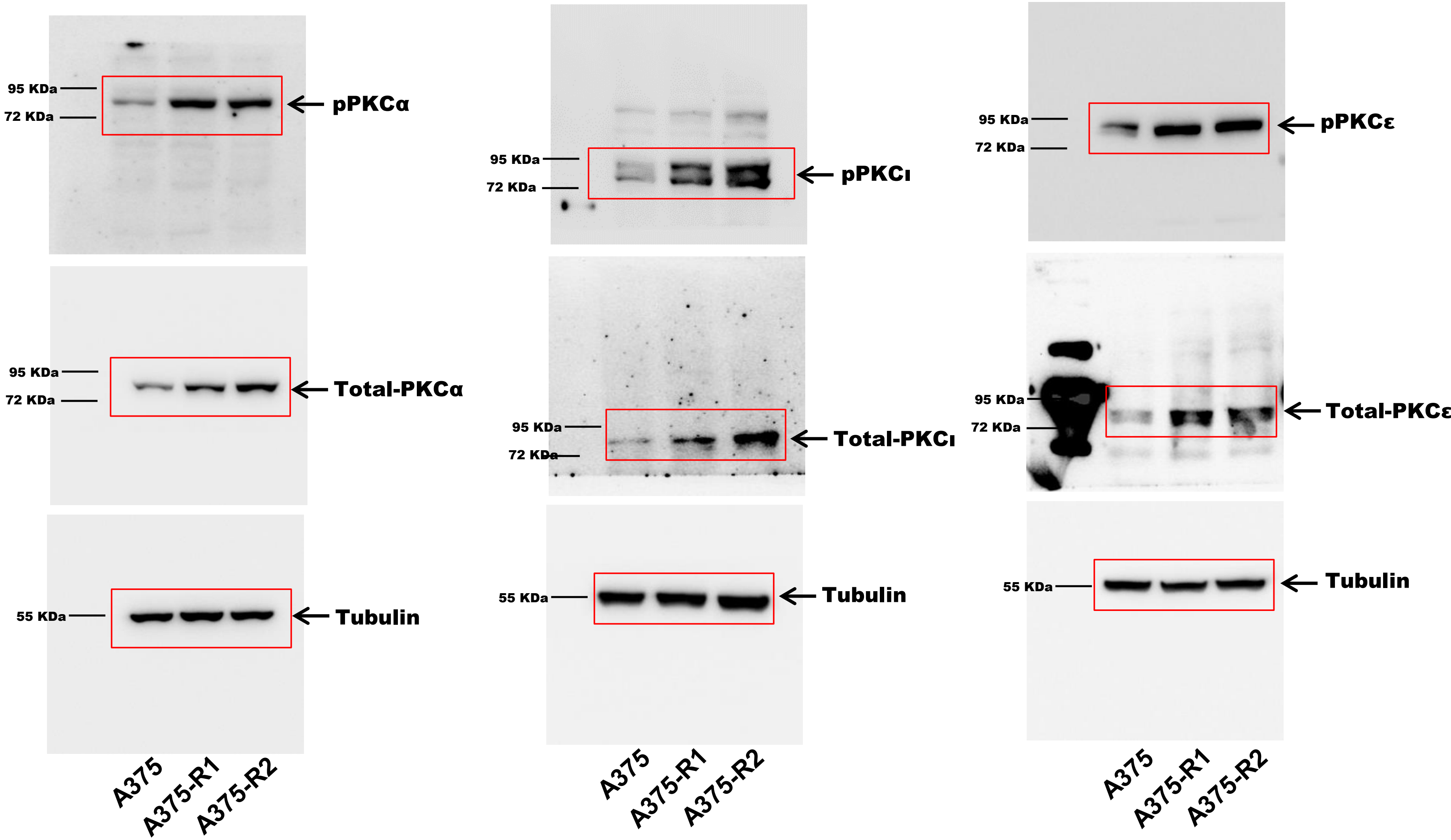

95 KDa
72 KDa
pPKCα
Total-PKCα
55 KDa
Tubulin
pPKCι
Total-PKCι
pPKCε
Total-PKCε
A375
A375-R1
A375-R2

Figure 7C

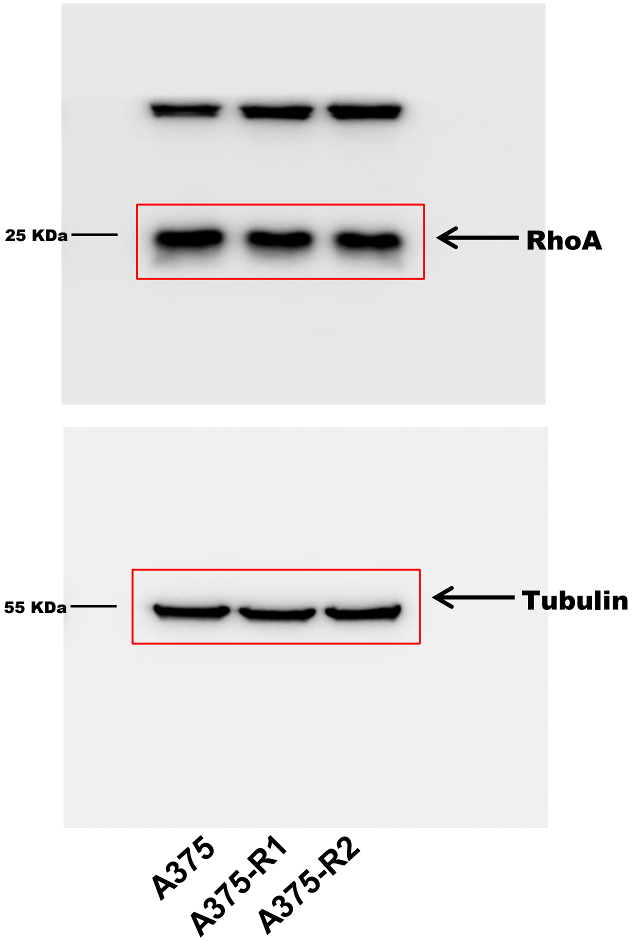

Figure 8B

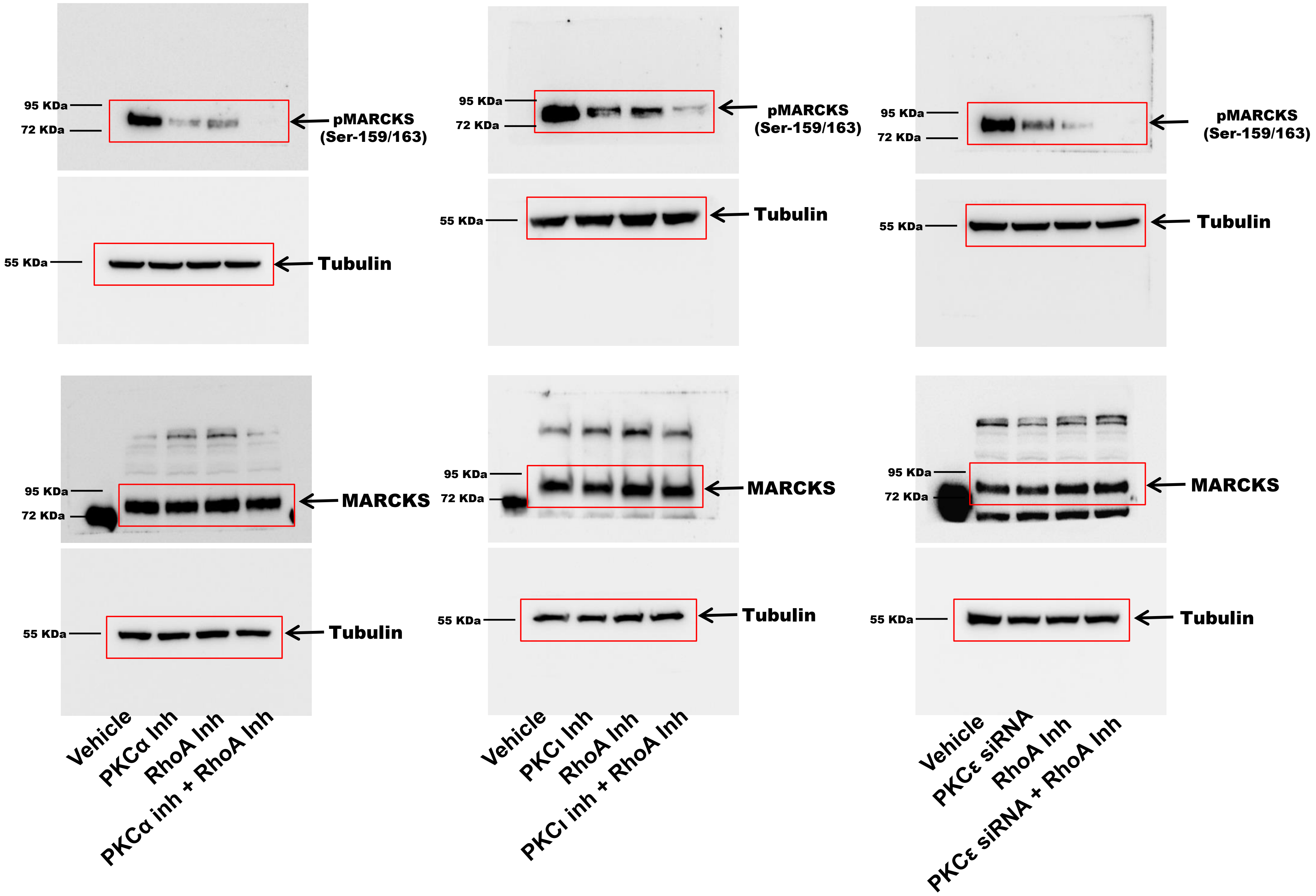
95 KDa
72 KDa
pMARCKS
(Ser-159/163)
55 KDa
Tubulin
95 KDa
72 KDa
MARCKS
55 KDa
Tubulin
Vehicle
PKCα Inh
RhoA Inh
PKCα inh + RhoA Inh
95 KDa
72 KDa
pMARCKS
(Ser-159/163)
55 KDa
Tubulin
95 KDa
72 KDa
MARCKS
55 KDa
Tubulin
Vehicle
PKCι Inh
RhoA Inh
PKCι inh + RhoA Inh
95 KDa
72 KDa
pMARCKS
(Ser-159/163)
55 KDa
Tubulin
95 KDa
72 KDa
MARCKS
55 KDa
Tubulin
Vehicle
PKCε siRNA
RhoA Inh
PKCε siRNA + RhoA Inh

Supplementary Fig S3

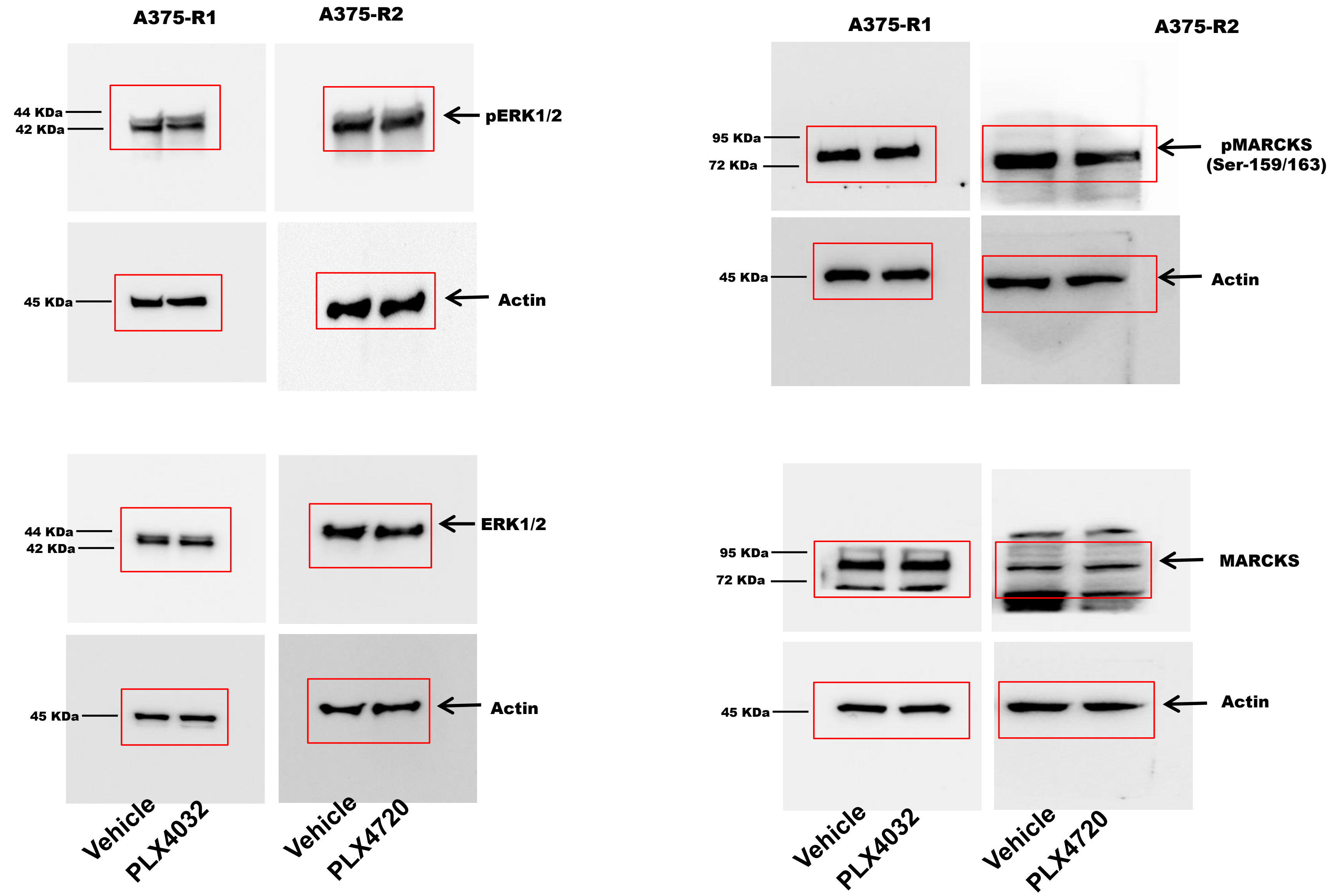

Supplementary Fig S4A

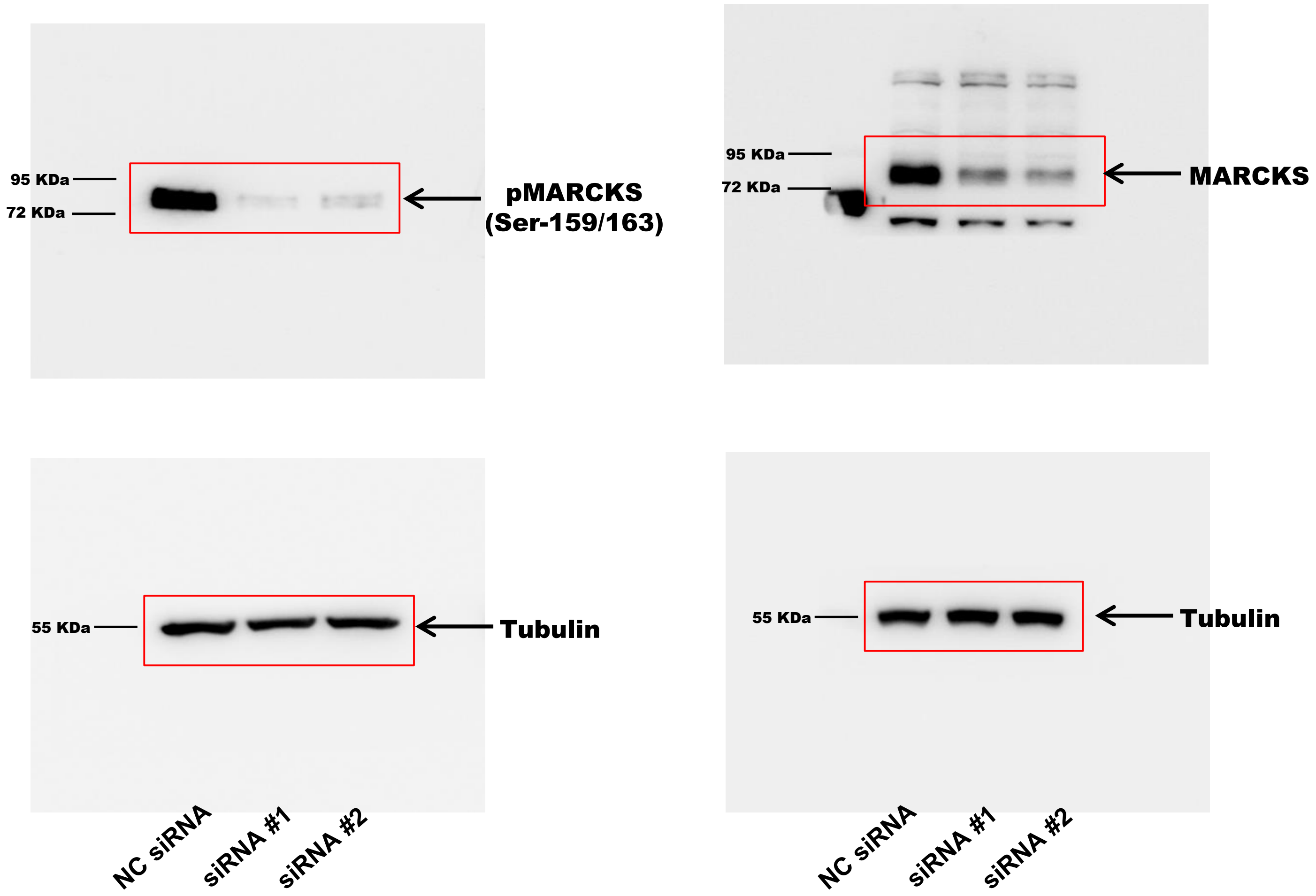

Supplementary Fig S7A

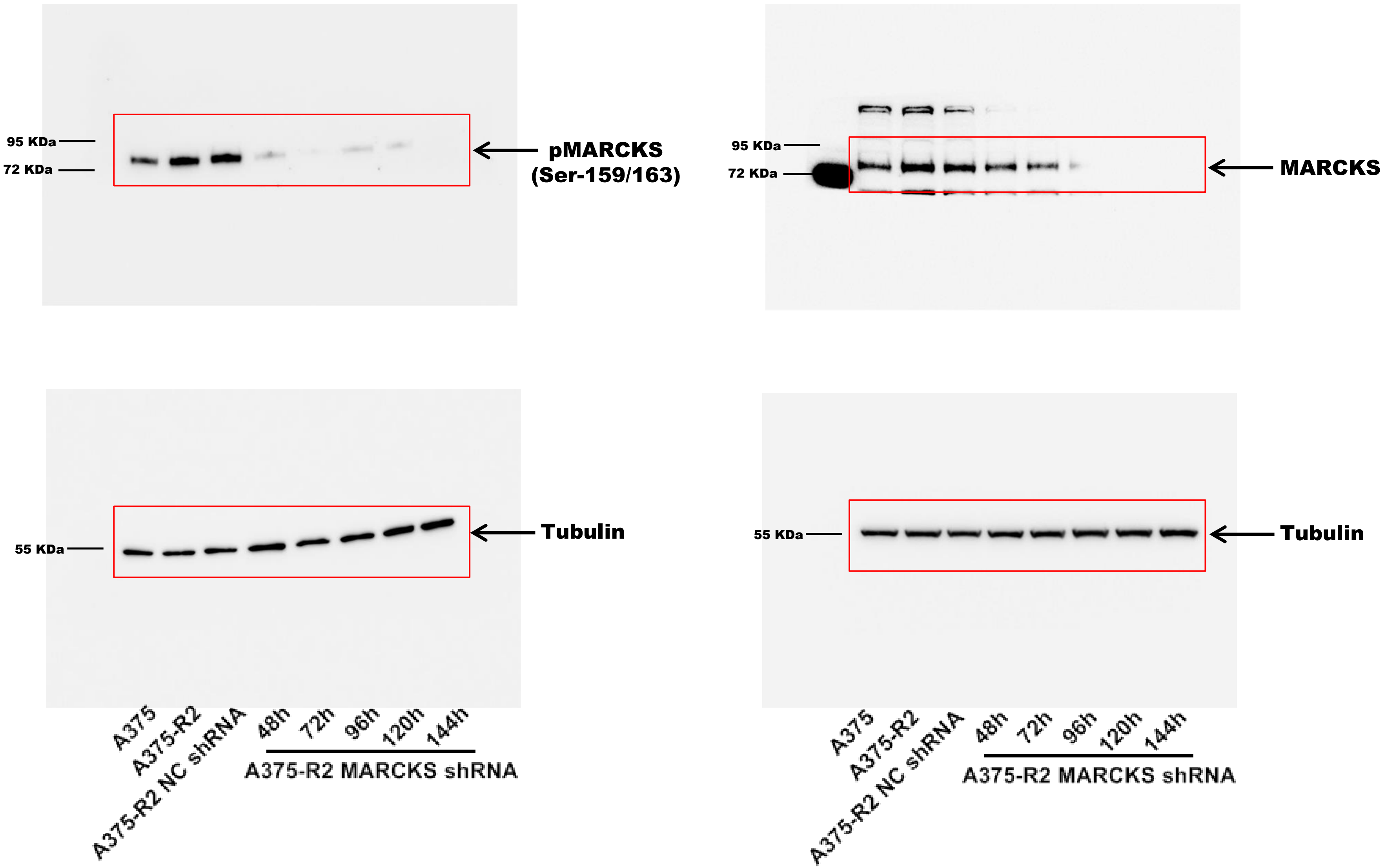

Supplementary Fig S8B

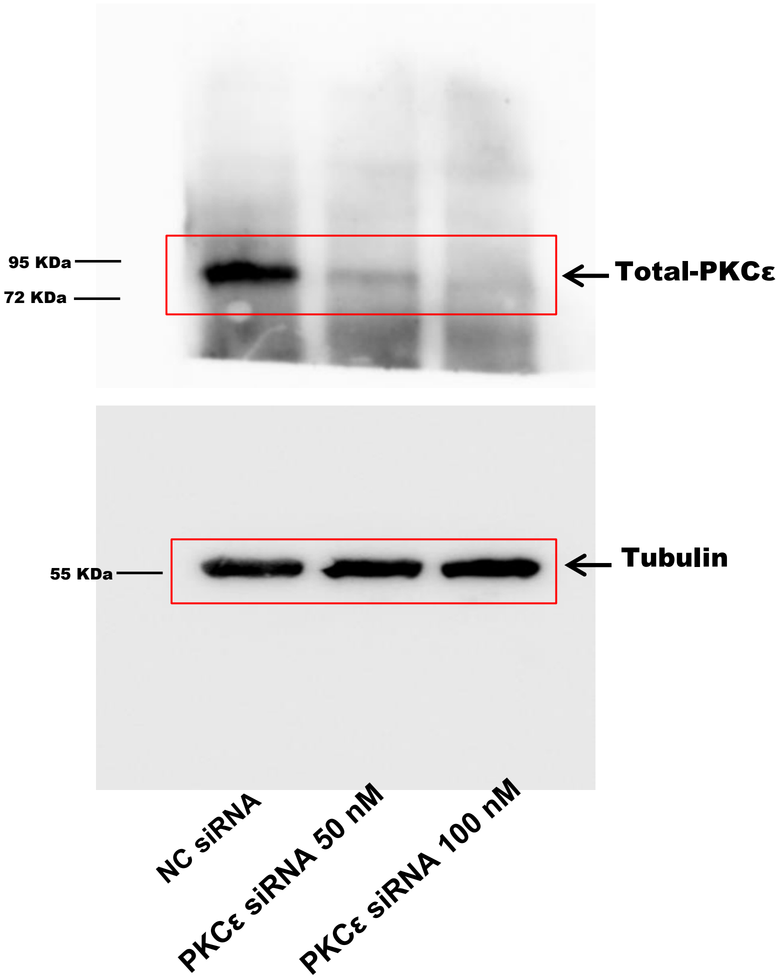

Supplement: Supplementary file 1 [file cancers-14-06077-s001.zip › File S1.pdf]
