# Supplementary material for: Increased MARCKS Activity in BRAF Inhibitor-Resistant Melanoma Cells Is Essential for Their Enhanced Metastatic Behavior Independent of Elevated WNT5A and IL-6 Signaling
Source: Cancers (Basel). 2022 Dec 10;14(24):6077. doi: 10.3390/cancers14246077 (PMC9775662; doi:10.3390/cancers14246077)
Supplement: Supplementary file 1 [file cancers-14-06077-s001.zip › Supplementary Figures S1-S8.pdf]

Supplementary Information

Increased MARCKS activity in BRAF inhibitor-resistant melanoma cells is essential for their enhanced metastatic behavior independent of elevated WNT5A and IL-6 signaling

Vikas Yadav [1, *], Njainday Jobe [1], Shakti Ranjan Satapathy [1], Purusottam Mohapatra[1,2] and Tommy Andersson [1, *]

[1] Cell and Experimental Pathology, Department of Translational Medicine, Lund University, Clinical Research Centre, Skåne University Hospital, SE 20213 Malmö, Sweden.

[2] Present Address: Department of Biotechnology, National Institute of Pharmaceutical Education & Research (NIPER), Guwahati, 781101, Assam, India

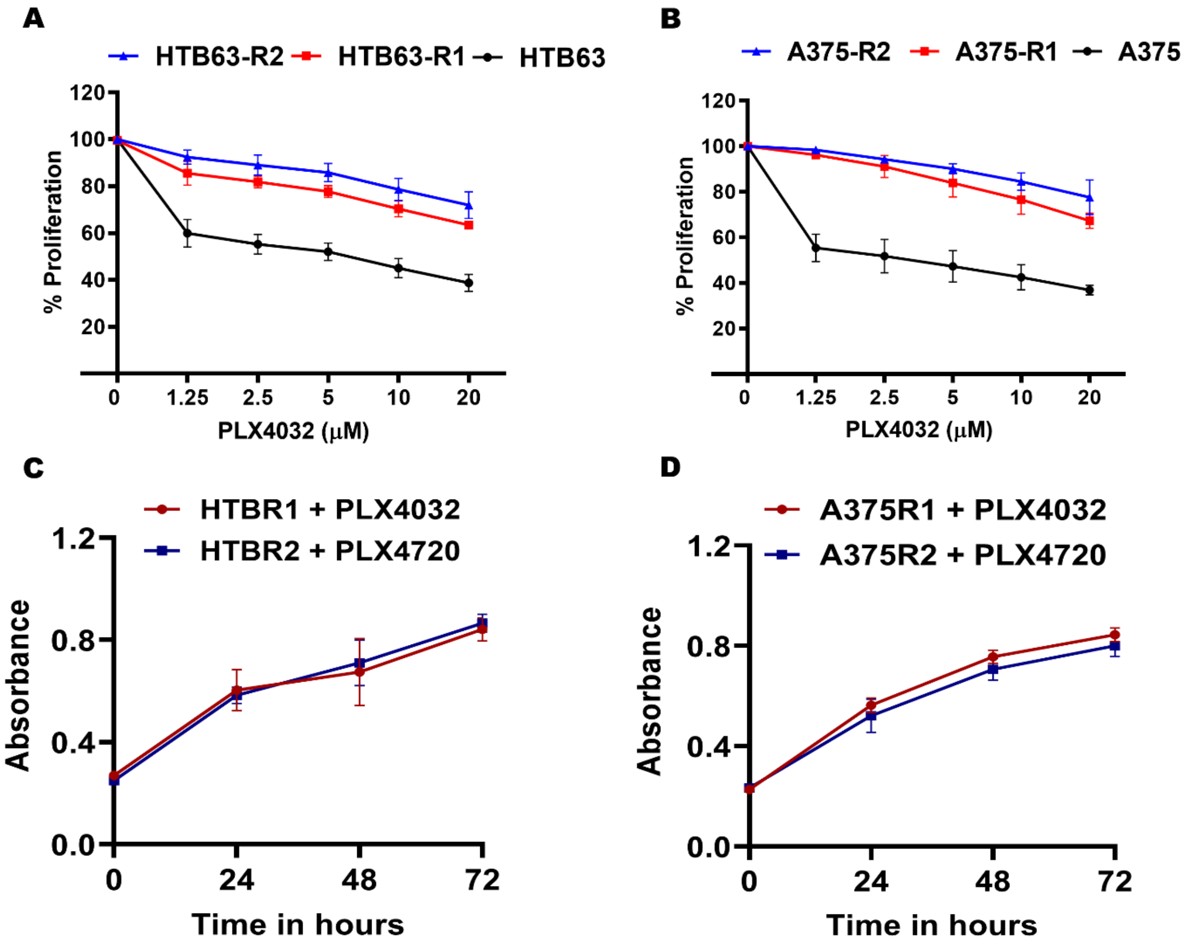

Supplementary Figure S1. WST1 cell proliferation assays were performed to evaluate the development of BRAFi resistance to PLX4032 **(A, B)** in melanoma cells as described in the "Materials and methods" section. BRAFi-sensitive (black circle), PLX-4032-R (R1; red square), and PLX-4720-R (R2; blue triangle) cells were exposed to increasing concentrations of BRAF inhibitors for 72 h. Graphs were generated from 4 independent experiments, and values are presented as the means (n=4) ± SEMs. The IC_{50} values for PLX4032 treatment were as follows: 5 μM for A375 BRAFi-sensitive, 5 μM for HTB63 BRAFi-sensitive, and >20 μM for A375-R1, A375-R2, HTB63-R1, and HTB63-R2 cells. **(C, D)** WST1 cell proliferation curves showing the response of established cell lines response in the presence of their respective BRAFi.

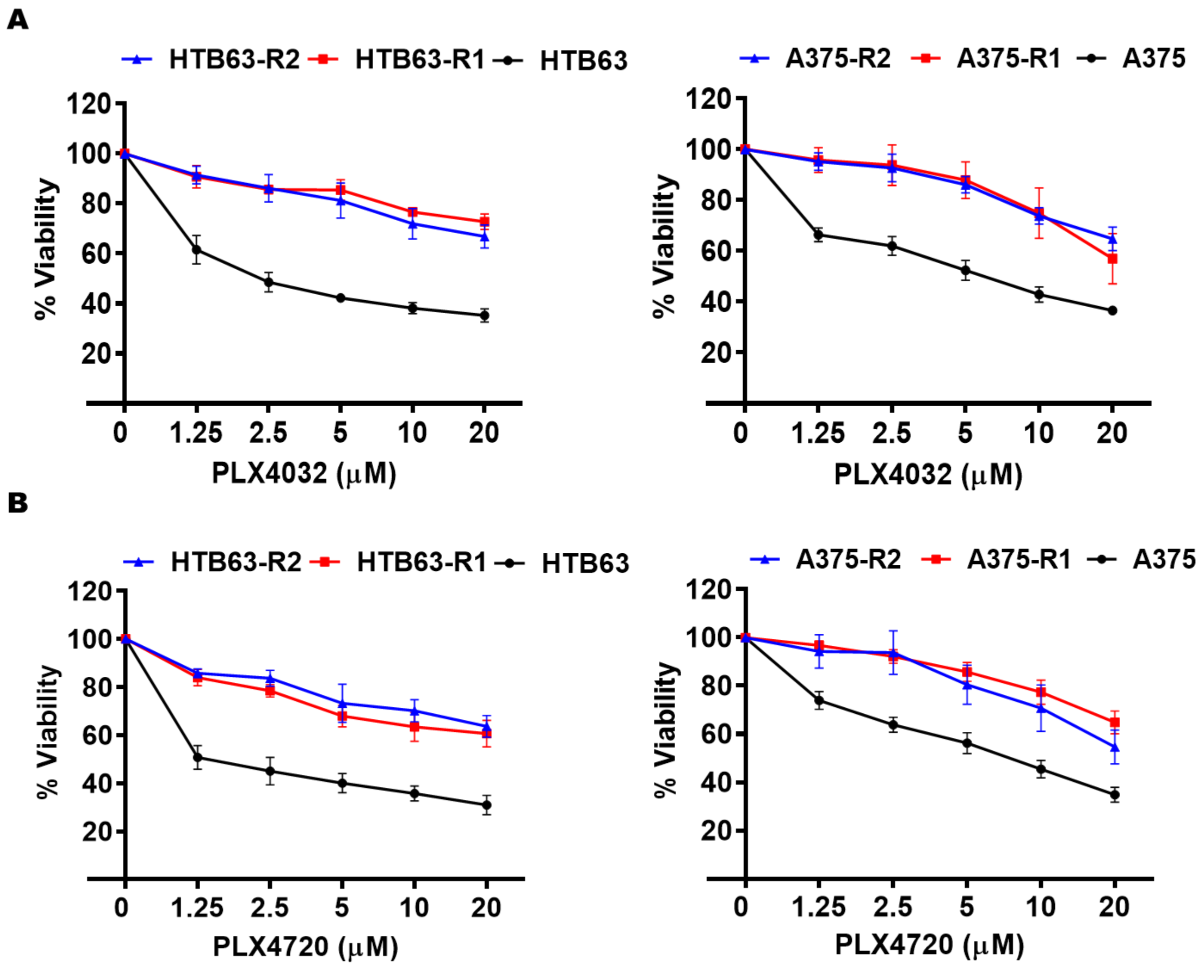

Supplementary Figure S2. MTT assays were performed to evaluate the development of BRAFi resistance to PLX4032 **(A)** and PLX4720 **(B)** in melanoma cells as described in the "Materials and methods" section. BRAFi-sensitive (black circle), PLX-4032-resistant (R1; red square), and PLX-4720-resistant (R2; blue triangle) cells were exposed to increasing concentrations of BRAF inhibitors for 72 h. Graphs were generated from 4 independent experiments, and values are presented as the means (n=4) ± SEMs. The IC_{50} values for PLX4032 treatment were as follows: 5 µM for A375 BRAFi-sensitive, 2.5 µM for HTB63 BRAFi-sensitive, and >20 µM for A375-R1, A375-R2, HTB63-R1, and HTB63-R2 cells. The IC_{50} values for PLX4720 treatment were as follows: 7.5 µM for A375 BRAFi-sensitive, 1.25 µM for HTB63 BRAFi-sensitive, and >20 µM for A375-R1, A375-R2, HTB63-R1, and HTB63-R2 cells.

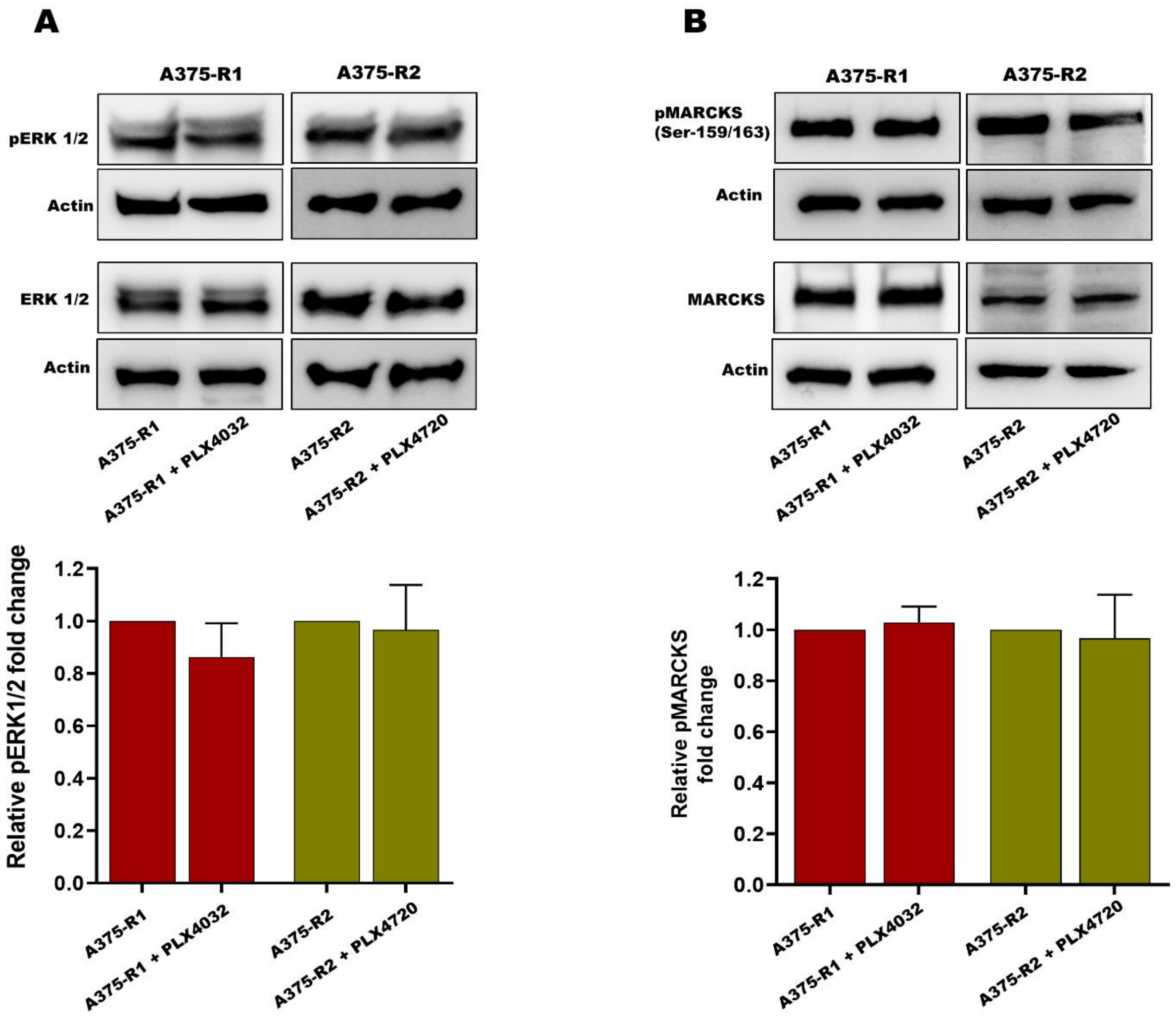

Supplementary Figure S3. Western blot analysis showing the levels of pERK1/2, total ERK **(A),** and pMARCKS (Ser-159/163), total MARCKS **(B)** in BRAFi-R A375-R1 and A375-R2 cells when treated with their respective BRAFi. Representative blots from 4 independent experiments are shown with actin as the loading control. Graphs below the blot shows the densitometric analysis of pERK1/2 and pMARCKS in BRAFi-R A375-R1 and A375-R2 cells when treated with their respective BRAFi. The obtained values were normalized to the value of the actin loading control from the same sample. The calculated pMARCKS/actin, pERK1/2/actin ratios were normalized to those in the corresponding vehicle treated and are presented as relative fold changes.

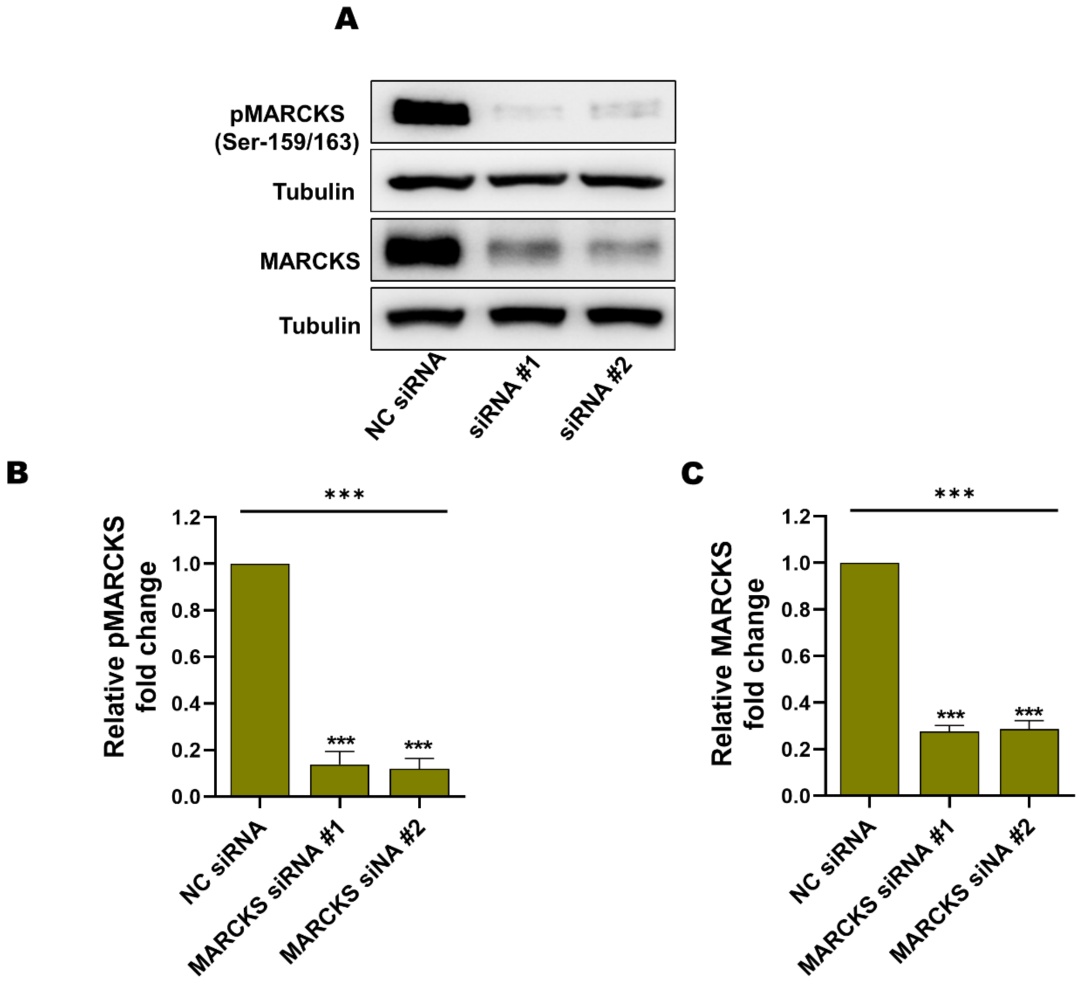

Supplementary Figure S4. (A) Western blot analysis showing the levels of pMARCKS (Ser-159/163) and total MARCKS in A375-R2 cells transfected with negative control siRNA (NC; 50 nM) or either of two different MARCKS-targeting siRNAs (50 nM). Representative blots from 4 independent experiments are shown with tubulin as the loading control. **(B, C)** Graphs showing the densitometric analysis of the pMARCKS (Ser-159/163) **(B)** and total MARCKS levels **(C)** in A375-R2 cells transfected with NC siRNA or either of two different MARCKS-targeting siRNAs. The calculated pMARCKS/tubulin and total MARCKS/tubulin ratios were normalized to those in the corresponding negative control (NC) siRNA-transfected cells and are presented as relative fold changes. The data were calculated from 4 independent experiments, and the results are given as the means ± SEMs. Statistical significance was estimated using ANOVA with Dunnett’s post hoc test for multiple comparisons; ** $p<0.01$, *** $p<0.001$.

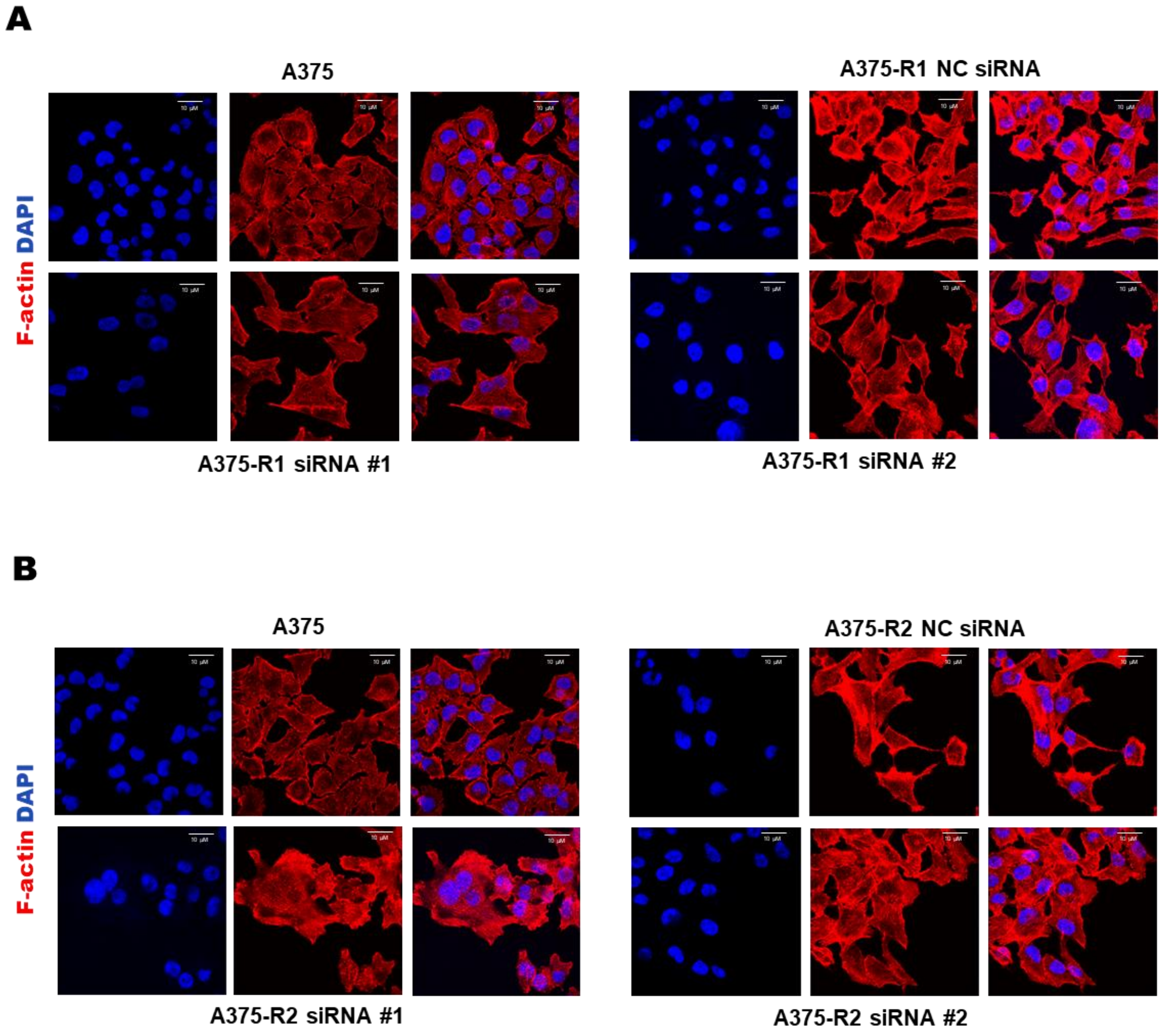

Supplementary Figure S5. A375-R1 (A) and A375-R2 (B) melanoma cells were transfected with negative control siRNA (NC; 50 nM) or either of two different MARCKS-targeting siRNAs (50 nM), after which they and A375 cells were stained with phalloidin-TRITC (F-actin) and DAPI as described in the “Materials and methods” section. The representative images correspond to Figure 5. These images are at 40X magnification and the scale bars represent 10 µm.

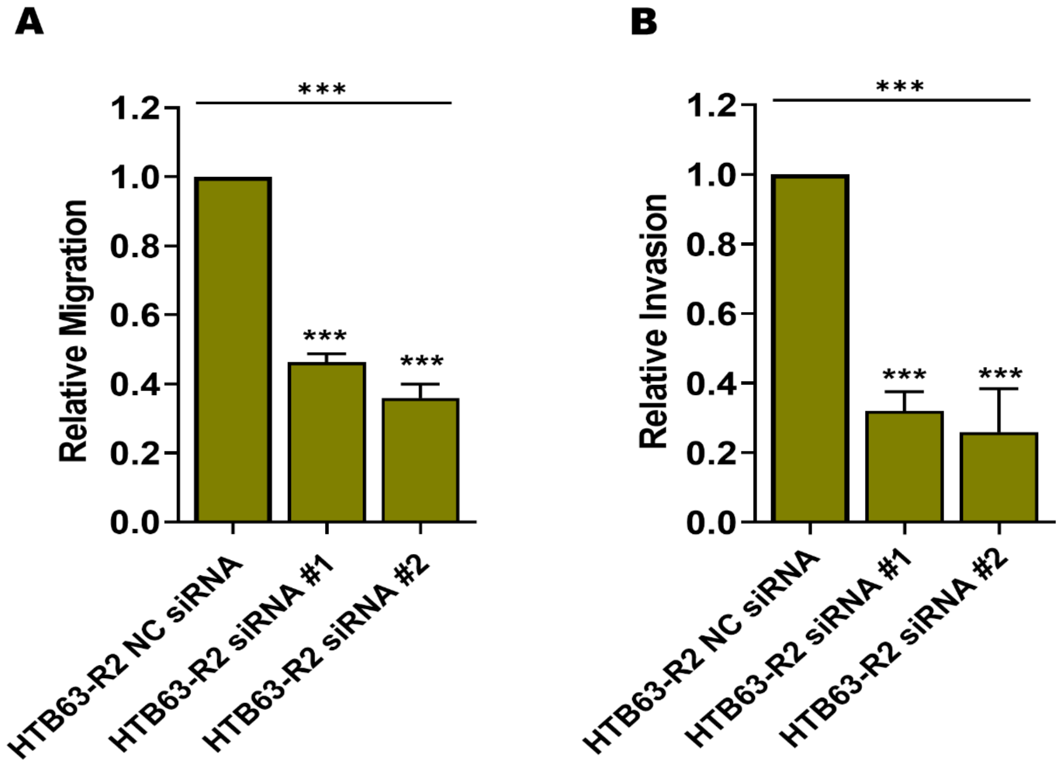

Supplementary Figure S6. Graphs showing the migration **(A)** and invasion **(B)** capacities of HTB63-R2 melanoma cells transfected with control siRNA (NC; 50 nM) or either of two different MARCKS-targeting siRNAs (50 nM). The numbers of migrated and invaded cells were determined using NIH ImageJ software, and the values were normalized to those in NC siRNA-transfected cells and are given as the mean ± SEM of 4 independent experiments. Statistical significance was estimated using ANOVA with Dunnett's *post hoc* test for multiple comparisons; *** $p<0.001$.

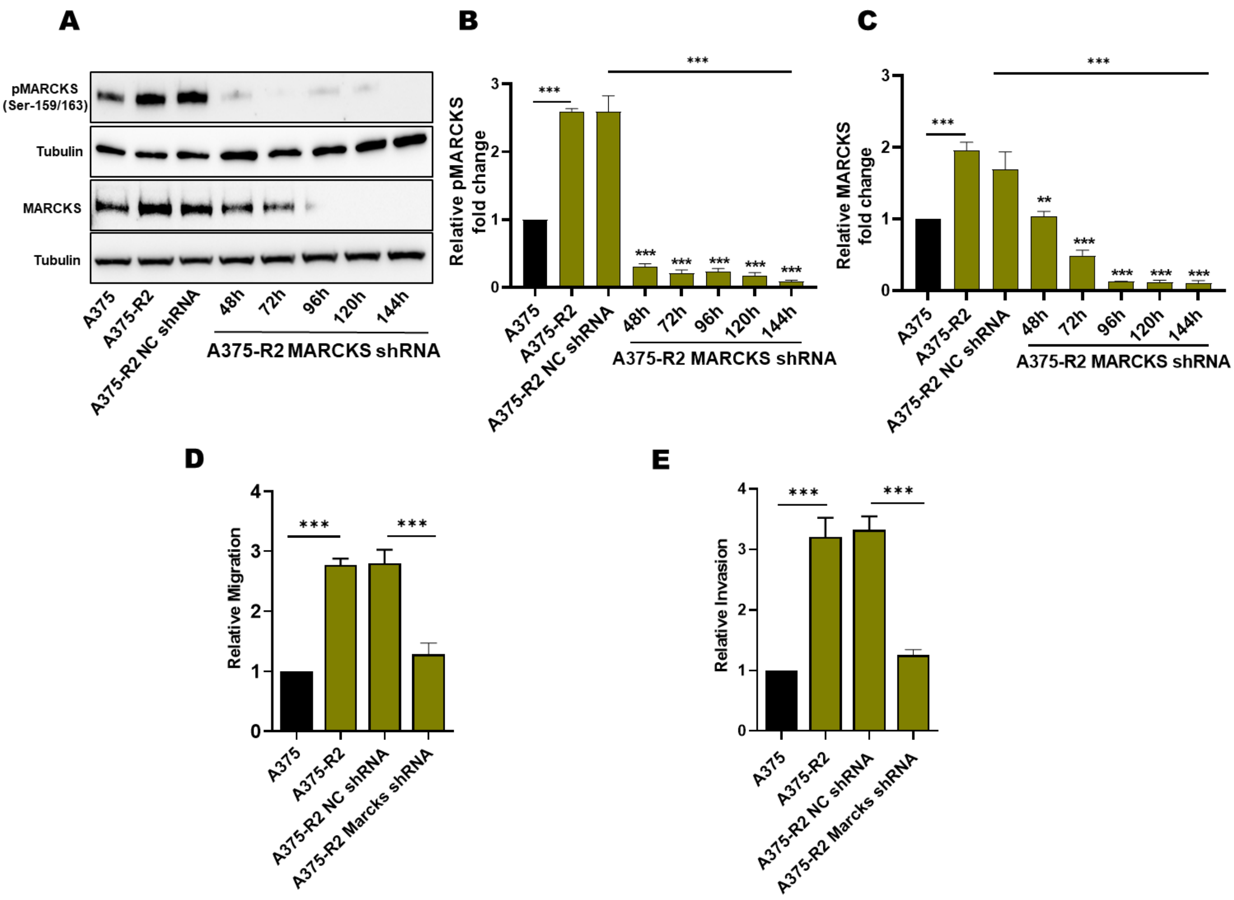

Supplementary Figure S7. (A) Western blot analysis showing the levels of pMARCKS (Ser-159/163) and total MARCKS in A375 BRAFi-sensitive cells and in non-transfected BRAFi-R A375-R2 cells as well as in BRAFi-R A375-R2 cells transfected with negative control shRNA (NC) or MARCKS-targeting shRNA. The latter group was analyzed over a time course. Representative blots from 4 independent experiments are shown with tubulin as the loading control. **(B, C)** Graphs showing the densitometric analysis of the pMARCKS (Ser-159/163) **(B),** and total MARCKS levels **(C)** in A375 BRAFi-sensitive cells and in non-transfected BRAFi-R A375-R2 cells as well as in BRAFi-R A375-R2 cells transfected with negative control shRNA (NC) or MARCKS-targeting shRNA. The latter group was analyzed over a time course. **(D, E)** Transwell-based migration and invasion assays were performed with the above groups of cells to evaluate their migration **(D)** and invasion **(E)** capacities. The numbers of migrated and invaded cells were determined using NIH ImageJ software, and the values were normalized to those in BRAFi-sensitive melanoma cells and are given as the mean ± SEM of 4 independent experiments. Statistical significance was estimated using ANOVA with Dunnett’s *post hoc* test for multiple comparisons; ** $p<0.01$, *** $p<0.001$. The time-dependent effects of MARCKS shRNA were statistically analyzed by comparison with negative control shRNA-transfected cells.

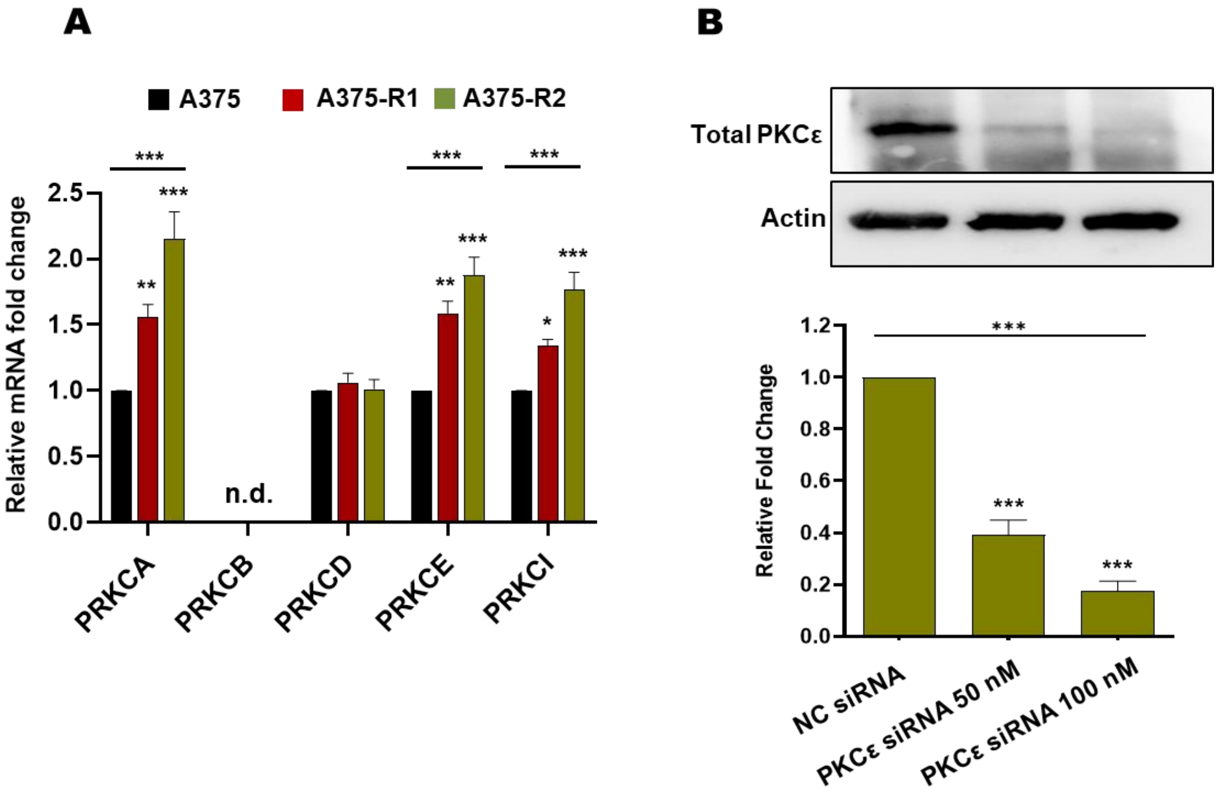

Supplementary Figure S8. (A) Graph showing the relative mRNA expression of PRKCA, PRKCB, PRKCD, PRKCE, and PRKCI in BRAFi-sensitive, A375-R1 and A375-R2 cells. The mRNA fold change was calculated using MxPro software. Graphs were generated from 4 independent experiments; relative expression is presented as fold changes, with the values given as the means ± SEMs. Statistical significance was estimated using ANOVA and Tukey's *post hoc* test for multiple comparisons; n.d. = not detectable/no C_T values, * $p<0.05$, ** $p<0.01$, *** $p<0.001$. **(B)** Western blot analysis showing the levels of total PKCε in A375-R2 cells transfected with negative control siRNA (NC; 100 nM) or PKCε-targeting siRNAs (50 and 100 nM). Representative blots from 4 independent experiments are shown with actin as the loading control. Graph below the blot shows its respective densitometric analysis. The calculated PKCε/actin ratios were normalized to those in the corresponding negative control (NC) siRNA-transfected cells and are presented as relative fold changes. The data were calculated from 4 independent experiments, and the results are given as the means ± SEMs. Statistical significance was estimated using ANOVA with Dunnett's post hoc test for multiple comparisons; *** $p<0.001$
